# Proceedings of the 1st Liaquat University of Medical & Health Sciences (LUMHS) International Medical Research Conference

**DOI:** 10.1186/s40001-017-0296-3

**Published:** 2017-12-28

**Authors:** Mohsin Iqbal, Marie-Veronique Clement-Pervaiz, Muhammad Jaffer Ansari, Shazib Pervaiz, Salma Sheikh, Shahjahan Katpar, Sultan Ayoub Meo, Kamran Sattar, Susie Schofield, Ahmet Kağan Karabulut, Amir Iqbal Memon, Farah Naz Memon, Hafeez Ahmed, Aneela A. Rahman, Ikram Din Ujjan, Mishal Ahmed, Javed Altaf, Muhammad Adeel Mahesar, Taimur Jatoi, Jewat Sunder, Satti Jewat, Aziz Memon, Hinna Feroz, Roomi Aijaz, Kolachi Hussain Bux, Muhammad Imran Rathore, Samreen Memon, Pushpa Goswami, Jaweria Samejo, Mona Humaira, Kashif Zakria, Razia Hanif Ghani, Hanif Ghani, Shoaib Ansari, Muhammad Akbar Nizamani, Jan Muhammad Memon, Khalid Iqbal Talpur, Inayat Ullah Memon, Khuda Bux Mangrio, Shahzad Shaikh, Mahesh Kumar, Yasir Arafat, Naveera Fatima, Mariya Qazi, Syed Fasih Ahmed Hashmi, Muhammad Ali Bohyo, Seema Bibi, Raheel Sikundar, Yaqoob Shahani, Ali Muhammad Waryah, Umbreen Bano, Pashmina Sheikh, Samina Gul, Naveera Rafique, Samina Memon, Sobia Wali Muhammad, Yasir Arfat Memon, Shehzad Sheikh, Muhammad Khalid Shaikh, Gulshad Wagan, Pushpa Chetan Das, Sana Zahiruddin, Neeta Sham, Nigar Jabeen, Sanam Maree, Binafsha Manzoor Syed, Bikha Ram Derajani, Altaf Talpur, Sharjeel Abbas, Abdul Ghaffar Memon, Adeel Abbas, Madiha Iqbal, Waseem Riaz, Mohsin Hussain, Fahmeena Qadri, Abdul Rehman Shaikh, Arshi Naz, Abdullah Kamran Soomro, Doulat Bajaj, Shaista Shah, Muhammad Asif Syed, Aneela Atta Ur Rahman, Tahir Sultan Shamsi, Aijaz Qadir Patoli, Nusrat Sehto, Shaista Aijaz, Aisha Arshad, Samina Naz Mukry, Madiha Saud, Iffat Shamim, Muhammad Nadeem, Tahir Shamsi, Ammar Hameed Khan, Muhammad Muneeb, Asra Talpur, Farzana Chang, Furqan Ahmed Bhatti, Sadia Effendi, Fahad Ahmed Memon, Khalida Naz Memon, Pashma Memon, Gulzar Usman, Bilal Razzaq Memon, Faheem Ahmed Memon, Faiza Memon, Aneela Atta Ur Rahmaan, Muhammad Ilyas Siddiqui, Faiza Shabir Ahmed, Feriha Fatima, Farhana Rajpar, Farheen Shaikh, Muhammad Yousuf Memon, Tazeen Shah, Shafaq Ansari, Fayaz Hussian Mangi, Jawaid Naeem Qureshi, Naeem Ahmed Laghari, Fiza Shah Syed, Madiha Shah, Sanam Pahnwar, Hina Riaz, Zulfiqar Laghari, Suleman pirzada, Hina Shaikh, Shaikh Jeeaindo, Hidayatullah Mahesar, Naem Tarique Narejo, Maria Jawed Badvi, Jawed Ahmed Badvi, Kulsoom Jawed, Mohsin Iqbal Haroon, Nadia Khan, Naheed Perveen, Naveena Fatima, Munira Borhany, Nida Anwar, Imran Naseer, Rehan Ansari, Samson Boota, Mustansir Zaidi, Nazia Hafeez, Faheem A. Memon, Parveen Akhtar, Zanab Khatoon, Miss Vectoria, Ghulam Abass, Rafeen Talpur, Rafiq Ahmed, Roohi Naz, Ali Raza Memon, Zainab Memon, Raheela Munwar, Sajida Rajpar, Fareen Memon, Miss Bilquees, Razia Shoukat, Sadia Abbasi, Sadia Shahmeer Qazi, Sama paras, Sana Fatima, Abdul Rehman Khalil Shaikh, Sana Zaheeruddin, Shahid Memon, Mahjabeen Shaikh, Sara Khalid Memon, Sidra Qadir, Sumaira Shaikh, Syed Fasih Ahmad, Zeeshan Nasir, Syna Pervaiz Singha, Afroz Saleem Kazi, Usha Isaac, Tanweer Ahmed Shaikh, Tarachand Devrajani, Syed Zulfiquar Ali Shah, Samar Raza, Urooj Bhatti, Tarim Nayab, Nehan Syed, Yar Muhammad Waryah, Uzma Zaidi, Saba Shahid, Naveen Fatima, Sharik Ahmed, Gul Safaida

**Affiliations:** 1 Shaheed Zulfiqar Ali Bhutto Medical University, Islamabad, Pakistan; 2 Department of Biochemistry, Yong Loo Lin School of Medicine, NUS Graduate School for Integrative Science and Engineering, National University of Singapore, Singapore, Singapore; 3 Washington University, Barnes Jewish Health Care, Memorial Hospital, Belleville, IL USA; 4 Department of Physiology, Yong Loo Lin School of Medicine and NUS Graduate School for Integrative Sciences and Engineering, National University of Singapore, Singapore, Singapore; 5 National University Cancer Institute, NUHS, Singapore, Singapore; 6 Liaquat University of Medical & Health Sciences, Jamshoro, Pakistan; 7 Institute of Dentistry, LUMHS, Jamshoro, Pakistan; 8 College of Medicine, King Saud University, Riyadh, Saudi Arabia; 9 Centre for Medical Education, Dundee, UK; 10 Faculty of Medicine, Selcuk University, Konya, Turkey; 11 Department of Urology, Liaquat University of Medical and Health Sciences, Jamshoro, Pakistan; 12 Sindh United (n) Developmental Educational Rural Society, Rotary Club Khipro, Khipro, Sindh Pakistan; 13 Department of Radiology, AKMCC, Hyderabad, Pakistan; 14 Department of Gynae, LUMHS, Jamshoro, Pakistan; 15 Isra University, Hyderabad, Pakistan; 16 Department of Anatomy, Muhammad Medical College, Mirpur Khas, Hyderabad, Pakistan; 17 Department of Anatomy, Liaquat University of Medical & Health Sciences, Jamshoro, Pakistan; 18 Department of Cardiology, LUHHS, Jamshoro, Pakistan; 19 Department of Medicine, LUMHS, Jamshoro, Pakistan; 20 Indus Medical College, Tando Muhammad Khan, Hyderabad, Pakistan; 21 Sindh Institute of Ophthalmology and Visual Sciences (SIOVS), Hyderabad, Pakistan; 22 Qasimabad, Hyderabad, Sindh Pakistan; 23 Department of Plastic Surgery, LUMHS, Jamshoro, Pakistan; 24 Department of Gynae and Obs, Liaquat University Hospital, Hyderabad, Sindh Pakistan; 25 Plastic Surgery Department, Liaquat University of Medical & Sciences, Jamshoro, Pakistan; 26 Department of Dermatology, LUMHS, Jamshoro, Pakistan; 27 Liaquat University Hospital, Hyderabad, Pakistan; 28 Shearwave Elastography Unit, Medical Research Centre, Liaquat University of Medical & Health Sciences, Jamshoro, Pakistan; 29 Department of Surgery, Liaquat University of Medical & Health Sciences, Jamshoro, Pakistan; 30 Department of Pathology, LUMHS, Jamshoro, Sindh Pakistan; 31 Diagnostic and Research Lab, Hyderabad, Pakistan; 32 Department of Community Health Sciences, Liaquat University of Medical and Health Sciences, Jamshoro, Pakistan; 33 Department of Clinical Hematology, National Institute of Blood, Disease Center and Bone Marrow Transplantation, Karachi, Pakistan; 34 Sir C.J. Institute of Psychiatry, Hyderabad, Pakistan; 35 Government Hospital, Qasimabad, Pakistan; 36 National Institute of Blood Diseases, Karachi, Pakistan; 37 Punjab Institute of Cardiology, Lahore, Pakistan; 38 Department of Community Medicine, LUMHS, Jamshoro, Pakistan; 39 Diagnostic and Research Laboratory, LUMHS, Hyderabad, Pakistan; 40 Faculty of Community Medicine & Public Health Sciences, LUMHS, Jamshoro, Pakistan; 41 Liaquat University Hospital, Jamshoro, Pakistan; 42 Department of Biochemistry, Liaquat University of Medical and Health Sciences, Jamshoro, Sindh Pakistan; 43 Department of Physiology, Liaquat University of Medical and Health Sciences, Jamshoro, Sindh Pakistan; 44 Department of Anesthesiology, Liaquat Medical Hospital, Jamshoro, Sindh Pakistan; 45 Nuclear Institute of Medicine and Radiotherapy, Jamshoro, Pakistan; 46 Medical Research Centre, Liaquat University of Medical & Health Sciences, Jamshoro, Pakistan; 47 Sindh University, Jamshoro, Pakistan; 48 MBGD, LUMHS, Jamshoro, Pakistan; 49 Department of F.W.B & Fisheries, University of Sindh, Jamshoro, Pakistan; 50 Department of Physiology, University of Sindh, Jamshoro, Pakistan; 51 Ghulam Mohammad Mehar College, Sukkur, Pakistan; 52 Dow International Medical College, Karachi, Pakistan; 53 Department of Gynaecology & Obstetric Unit IV, Liaquat University of Medical and Health Sciences, Jamshoro, Pakistan; 54 Department of Blood Bank of National Institute of Blood Disease and Bone Marrow Transplantation (NIBD), Karachi, Pakistan; 55 Diagnostic and Research Lab, Jamshoro, Pakistan; 56 People Nursing School, Jamshoro, Pakistan; 57 Department of Gynecology & Obstetrics, Indus Medical College Hospital, Tando Muhammad Khan, Sindh Pakistan; 58 National Institude of Blood Disease and Bone Marrow Transplantation, Karachi, Pakistan; 59 Department of Plastic & Reconstructive Surgery, L.U.M.H.S, Jamshoro, Pakistan; 60 National Institute of Blood Disease and Bone Marrow Transplantation, Karachi, Pakistan; 61 Agha Khan Hospital, Hyderabad, Pakistan; 62 Department of Sardiology, Liaquat University Hospital, Hyderabad, Pakistan; 63 Department Cardiology, Liaquat University of Medical and Health Sciences, Hyderabad, Pakistan; 64 Molecular Biology & Genetic Diseases Department, LUMHS, Jamshoro, Pakistan; 65 Department of Clinical Haematology, National Institute of Blood Diseases & Bone marrow Trasplantation, Kharachi, Pakistan; 66 Department of Genomics, National institute of Blood Diseases & Bone marrow Trasplantation, Kharachi, Pakistan; 67 Department of Research & Molecular Medicine, National Institute of Blood Diseases & Bone marrow Trasplantation, Kharachi, Pakistan

## Speaker presentations

### S1 Molecular genetics and functional studies of hearing impairment in Pakistani population

#### Mohsin Iqbal

##### Shaheed Zulfiqar Ali Bhutto Medical University, Islamabad, Pakistan

###### **Correspondence:** Mohsin Iqbal

*European Journal of Medical Research* 2017, **22(Suppl 1):**S1

Hereditary hearing impairment is one of the common monogenic disorder in Pakistan. It is estimated that around 0.16% peoples have moderate to profound prelingual hearing loss while one-third of people above 60 years have some sort hearing problem. I had enrolled 150 families with prelingual hearing loss. Initial screening of these families was done by genotyping reported loci with fluorescently labelled STR markers. Families showing linkage with specific loci was confirmed by NGS based platforms like OtoSeqand Sanger sequencing. Combination of these techniques led towards the identification. Combination of these techniques led towards the identification of 34 pathogenic variants in *GJB2, MYO7A, CDH23, MARVELD2* and *POU3F4* in 40 families. Fifteen of these variants have not reported earlier. Families remain unlinked with reported loci were subjected for Genome wide scan, SNP analysis and exome sequencing. A novel deafness locus *DFNB79* were mapped though GWS and three Novel gene *GIPC3, TAPRN* and *NARS2* were identified by the combination of Linkage analysis and next generation exome sequencing. Using NGS based platforms like OtoSeq, GWS, SNP analysis and exome sequencing in families segregating hearing loss, will contribute to the identification of common and population specific mutations, early diagnosis, genetic counseling and molecular epidemiology.

### S2 Superoxide and carcinogenesis: my 20 years’ obsession

#### Marie-Veronique Clement-Pervaiz

##### Department of Biochemistry, Yong Loo Lin School of Medicine, NUS Graduate School for Integrative Science and Engineering, National University of Singapore, Singapore

###### **Correspondence:** Marie-Veronique Clement-Pervaiz

*European Journal of Medical Research* 2017, **22(Suppl 1):**S2

Over the years work of our group dispelled the dogmatic view of reactive oxygen species as only toxic molecules by providing evidence for their involvement in cell survival signaling pertinent to carcinogenesis. Together our findings led us to propose superoxide (O_2_^·−^) as the key oxygen species involved in the process of carcinogenesis. In support for this proposal we demonstrated that an increase in the intracellular level of O_2_^.−^ led to the regulation of two important regulators of tumor survival and development, the survival kinase Akt and the Na+/H+ exchanger NHE-1. More recently, analysis of breast carcinomas from The Cancer Genome Atlas (TCGA) database revealed strong positive correlation between tumors’epithelial to mesenchymal (EMT) score and the expression of O_2_^·−^ regulator MnSOD. This positive correlation between MnSOD and EMT score was significant and consistent across all breast cancer subtypes. Interestingly, using phenotypically distinct breast cancer cell lines, we provided evidence that constitutively high or induced expression of MnSOD promotes the EMT-like phenotype by way of a redox milieu predominantly driven by hydrogen peroxide (H_2_O_2_). Conversely, gene knockdown of MnSOD results in the reversal of EMT to a MET-like program, which appears to be a function of superoxide (O_2_^·−^)-directed signaling. These data underscore the involvement of MnSOD in the regulation of the switch between the EMT and MET-associated phenotype by influencing cellular redox environment via its effect on the intracellular ratio of O_2_^·−^ and H_2_O_2_. My address I will present these new findings and discus their importance for the management of breast cancer.

### S3 Recent advances in the field of cardiology

#### Muhammad Jaffer Ansari

##### Washington University, Barnes Jewish Health Care, Memorial Hospital, Belleville, IL, USA

###### **Correspondence:** Muhammad Jaffer Ansari

*European Journal of Medical Research* 2017, **22(Suppl 1):**S3

I will discuss recent advances in the field of cardiology, and how the management of the patients has changed in the last decade. What are the recent advances in Interventional Cardiology? What is the future of research in Cardiology? What are the three most important advances in cardiology in the recent years which has impacted and revolutionized our clinical practice? How can USA contribute to improve the quality of research in Pakistan? How can technology help to improve the quality of care and research at LUMHS. I will give my suggestions to promote research and academic activities in the field of cardiology and medicine. What is the real unmet clinical need in cardiology for Pakistan? How can we focus the research for the improvement of quality of care in the right areas? We need ground breaking changes which can impact community, not just papers to publish. What are the challenges in inculcating the most advanced technology and state of the art therapies in Pakistan? I will finally discuss how to handle the financial, logistical, and procedural hurdles for research and practice of medicine in Sindh/Pakistan.

### S4 Redox regulation of cancer cell fate decisions

#### Shazib Pervaiz^1,2^

##### ^1^Department of Physiology, Yong Loo Lin School of Medicine and NUS Graduate School for Integrative Sciences and Engineering, National University of Singapore, Singapore; ^2^National University Cancer Institute, NUHS, Singapore

###### **Correspondence:** Shazib Pervaiz

*European Journal of Medical Research* 2017, **22(Suppl 1):**S4

The primary focus of our program over the years has been to investigate signaling networks that promote cellular transformation and elucidate mechanisms that endow cancer cells the ability to evade death execution. As such, research efforts have been dedicated to identifying potential bottlenecks or vulnerabilities that cut across a spectrum of human cancers, including hematopoietic malignancies and solid tumors involving the breast, colon, lung and pancreas. To that end, using a variety of model systems such as oncogene addiction-induced proliferation, overexpression of proteins that block apoptosis execution, pharmacological inhibition of key cellular metabolic regulators, receptor and chemotherapy-induced death stimuli, and novel small molecule compounds, we provide evidence that cellular redox metabolism critically impacts cell fate decisions. Of note, across a wide spectrum of cellular redox stress, there emerges a dichotomy of responses in terms of cell survival/proliferation and cell death. At the lower end of the scale cell survival and proliferation is favored, while at the other extreme cell execution is the preferred outcome. How these varied levels of stress evoke disparate biological responses with distinct functional outcome(s) and the signaling networks and potential cellular targets that could be amenable to redox modification(s) are the major focus of our ongoing and future investigations. A summary of our key findings and ongoing/future studies as well as their relevance and application to unraveling the signaling nodes and Achilles heel of cancer will be discussed.

### S5 Evidence based interventions to prevent intergenerational stunting

#### Salma sheikh

##### LUMHS, Jamshoro, Pakistan

###### **Correspondence:** Salma sheikh

*European Journal of Medical Research* 2017, **22(Suppl 1):**S5

Linear growth failure in childhood is the most prevalent form of undernutrition globally. An estimated 165 million children under 5 years of age are *stunted*, with a height-for-age Z-score (HAZ) below − 2. It has now been recognized as major global health priority and WHA targets to reduce stunting by 40% by 2025. Chronic malnutrition or Stunting in Pakistan is a National emergency with nearly half of children 44% being affected making it third highest for stunted children in the world. 9.6 million children suffer from nutritional deprivation in utero or early childhood mainly due to food insecurity, maternal malnutrition, frequent infections, inadequate health services and lack of awareness about feeding and health care practices. Pakistan needs to invest in Nutrition to break this cycle of malnutrition and poverty, with lack of serious commitment our country will face a generation of stunted and unproductive people in 15 years. As infants are entirely dependent on their mothers for nutrition during the first 500 days of life, and 70% brain forms in utero, improving maternal nutritional status is likely to improve fetal outcome. Indeed Prenatal multiple micronutrients has shown to reduce SGA by 9% and balanced energy and protein diet by 31%, in different trials. Studies show that daily iron supplementation during pregnancy reduces low birthweight by 20%. Other nutrition interventions in early infancy like exclusive breast feeding, appropriate complimentary feeding and micronutrient fortification or supplementation in first 2 years have shown to reduce stunting rates along with reduction in morbidity and mortality.

### S6 Transforming oral health academics & health care in Pakistan via introducing forensic odontology

#### Shahjahan Katpar

##### Institute of Dentistry, LUMHS, Jamshoro, Pakistan

###### **Correspondence:** Shahjahan Katpar

*European Journal of Medical Research* 2017, **22(Suppl 1):**S6

Forensic Odontology or Forensic Dentistry is an important and well established Dental Specialty in the Advanced World and its formal teaching has existed for more than a century. Time has come for this to be introduced in Pakistan for all its Academic, Research and Practical reasons pertaining to Justice System & Crime. Its significance in terms of reporting, sharing scientific evidence, handling Crime is influenced by the people who are properly Qualified and trained as Forensic Odontologists and unfortunately we have none. As our society is rapidly changing, becoming more commercial & losing its cultural, ethical & human touch, therefore we feel it is high time for this neglected Dental Specialty to be given birth-introduced as we aim for National Paradigm Shift and share this Presentation.

**Keywords:** Teeth, Oral cavity, Forensic odontology, Dentistry, Facial xrays, Trauma, Assault.

### S7 Science in Muslim world: where do we stand?

#### Sultan Ayoub Meo

##### College of Medicine, King Saud University, Riyadh, Saudi Arabia

###### **Correspondence:** Sultan Ayoub Meo

*European Journal of Medical Research* 2017, **22(Suppl 1):**S7

Muslim world covers an enormous geographic area, comprising of 57 countries with 1.82 billion people. Muslim scientists have a proud history of science during the period 750–1250 AD. The light of science extinguished from the Muslim world but it exists and blazed brightly elsewhere. Muslim countries especially the Arab states are rich in natural resources including oil, gas, gold, copper along with high income advantages. But, the science budget of wealthy states of the Organization of the Islamic Countries (OIC) is one of the lowest in the world, spending a frail 0.81% of their GDP on research and development (R&D). There are about 1900 universities and degree awarding institutes in Muslim states, out of them only 15% have published research papers in the indexed science journals. As per Shanghai Ranking 2015, only 10 universities achieved a place within top 500 universities in global science ranking. The science journals from the Muslim world are small in number and many of these journals do not have on-line access or indexed in major popular scientific databases. The majority of indexed journals are rotating around the impact factor 0.01–0.8. Muslim countries are producing only 10% research publications in ISI web of science indexed journal and have negligible percentage of patent registrations in US, Europe and Japan. Only two scientists one from 57 Muslim states won the Nobel Prize. Muslim countries must establish more universities and research institutes, create a scientific culture that recognizes the contribution of scientists and values higher education and research.

### S8 Professionalism in research; whose responsibility is it anyway?

#### Kamran Sattar

##### College of Medicine, King Saud University, Riyadh, Saudi Arabia

###### **Correspondence:** Kamran Sattar

*European Journal of Medical Research* 2017, **22(Suppl 1):**S8

This presentation provides a brief review of the Definitions, Principles, Purposes and Practices concerning professionalism in research. Professionalism in research demands adhering to principles of confidentiality, scientific/academic integrity, and accountability. However, literature provides evidence that the levels of misconduct appear to be higher than in the past. There lies a gap between ideals and reality existing in all aspects of research and we notice that the researchers. Researchers and the institutes have equal responsibility to create a culture where attributes of professionalism in research are maintained and If research is a professional activity, then more effort is needed to describe and establish clear standards for all that this professionalism implies.

### S9 Transforming medical education through collaborative research: laying down good foundations

#### Susie Schofield

##### Centre for Medical Education, Dundee, UK

###### **Correspondence:** Susie Schofield

*European Journal of Medical Research* 2017, **22(Suppl 1):**S9

Medical education encompasses education in the healthcare professions across the continuum of undergraduate, postgraduate and continuing education. It could be argued the Flexner Report of 1910 transformed both the nature and the process of medical education in America, establishing the biomedical model as the gold standard of medical training (Duffy 2011). A 100 years on all is not well. We see inequities in health care both within and between countries, health systems struggling to keep up with changes in demographics and epidemiological transitions, overflowing curricula for students and trainees, and overworked health professionals. At the same time there has been an increase in both international and transnational medicine, giving added challenges and opportunities. Within medical education there has been increased interest in the development of medical educators and educational methods employed. This has been driven by three interlinked themes: the professionalisation of medical education, increased accountability, and not only an increasing awareness of educational excellence but a need for research to provide best evidence for choices made (Harden et al. 1999; Swanwick 2008). Yet medical education research is often seen as the poor relation, frequently unfunded, single-centred, and under-valued by institutions as ‘serious papers’ for annual reviews (Todres et al. 2007). This keynote explores how we can encourage and support research collaborations between different institutions, nationally and internationally. What are the benefits to the researcher, the institution, the students and ultimately the nations’ health? How can we use those to drive forward collaborations? What theoretical models can we utilize? What are the barriers and how can we change these to challenges that can be solved? The talk will explore several important issues that should be addressed at an early stage of any collaborative research, ensuring good foundations on which to base current research and further collaborations. Throughout I will present a number of case studies, both from with the University of Dundee and from the literature. I finish by challenging the audience to think: what next? What part can each person at this conference play in this fascinating and rewarding area?

### S10 Whole rat embryo culture; as a tool for teratological screening

#### Ahmet Kağan Karabulut

##### Selcuk University Faculty of Medicine, Konya, Turkey

###### **Correspondence:** Ahmet Kağan Karabulut

*European Journal of Medical Research* 2017, **22(Suppl 1):**S10

Birth defects induced by maternal exposure to exogenous agents during pregnancy are preventable, if the agents themselves can be identified and avoided. The number of candidate chemicals or drugs for registration and authorization is increasing at a fast rate and only few of the existing substances have been tested for teratogenicity to date. Whole rat embryo culture (WEC) as a screening system for safety evaluation procedures was considered as promising alternative test for developmental toxicity and teratogenicity assays. Over the past 40 years, the WEC system has been developed, optimized, and validated as an in vitro teratogenicity assay. According to this method, the rat embryos were cultured in rotated culture bottles during the early organogenesis, typically over a 48 h period. The culture medium is supplemented with serum and specific percentages of oxygen over the culture period. Test compounds are added into the culture medium. At the end of the culture, embryos are examined and evaluated for abnormalities of developmental morphology and overall embryonic growth. To quantitate embryonic growth and development, six developmental stages of series of structures are defined and scored from 0 to 5. The total morphological score in combination with the other measurable evaluation parameters such as somite number, crown-rump length, yolk sac diameter and protein content reflects the degree of growth and differentiation of the embryo. Furthermore, teratologic doses of the test compounds can be evaluated. The WEC system is considered a well established in vitro model for identifying and characterizing teratogenic properties of test compounds. The successful validation of the procedure by the European Center for the Validation of Alternative Methods (ECVAM) in 1995, makes the WEC a complex in vitro embryotoxicity test with high accuracy and predictability.

## Oral presentations

### O1 Initial experience of single incision laparoscopic cholecystectomy with conventional instruments

#### Amir Iqbal Memon

##### Liaquat University of Medical & Health Sciences, Jamshoro, Pakistan

###### **Correspondence:** Amir Iqbal Memon

*European Journal of Medical Research* 2017, **22(Suppl 1):**O1

Single incision laparoscopic Surgery is now rapidly emerging approach to gall bladder disease. To observe the cosmetic results and cost effect of single incision laparoscopic cholecystectomy in selected cases. From January 2013 to December 2015—40 (80) female and 13 (26) male patients submitted for elective single incision cholecystectomy with conventional instruments. In all these patients, BMI were less than 30 kg/m^2^. Pneumoperitoneum was created, Keeping the pressure at the rate of 5–6 mm of hg. A peri-umbilical incision was made and two 5 and 10 mm trocar introduced separately, one for Telescope and two working ports. Standard rigid laparoscopic instruments are introduced along 10 mm with 30° telescope. Instrument clashes are avoided by chop stick technique of crossing them at a proximal point so that ends are away from each other the procedure was accomplished. Patients having acute cholecystitis and Hepatitis B & C were excluded from the study. Out of 53 (n = 106) 80 women (75.4%) and 26 men (24.5%) were enrolled in this study. Mean age was 44 years (ranging 18–70 years). Mean body index was 27 kg/m^2^ (ranges 18.5–35.5 kg/m^2^(2)). Mean operating time was 52.5 min (ranges 30–75 min). Out of 106 patients, six (5.6%) patients were converted into open method due to dense adhesion and two (1.8%) an extra port required for dissection. Single incision laparoscopic cholecystectomy can be safely performed with conventional instruments with minimal cost and better cosmetic results. The ideal candidates for the procedure are younger ones with lower BMI without the signs of acute inflammations.

### O2 Policies vs implementations in community based programs: everything happens for a reason: “a qualitative study to explore the gaps”

#### Farah Naz Memon, Hafeez Ahmed, Aneela A. Rahman, Ikram din Ujjan, Mishal Ahmed

##### Liaquat University of Medical & Health Sciences, Jamshoro, Pakistan

*European Journal of Medical Research* 2017, **22(Suppl 1):**O2

Community based programs are considered as cost effective ways to reduce disease burdens, however most of programs end up in development of new challenges. Rationale of this study was to explore the reasons for failure of such type of programs. “A qualitative study” was conducted to assess the policies and implementation strategies of two community based programs; Lady Health Workers Program (LHWP) and Community Midwives program (CMWP). Study population was divided into two categories; (A) Community health workers (LHWs, CMWs and LHSs) (B) Representatives of LHWP, MNCHP, WHO, UNFPA, UNICEF, DHMT and EDO Health. FGDs and IDIs were conducted where appropriate. “Constant Comparison Analysis” method was used. Two different programs with same goals and objectives with Lack of; commitment, linkages, ownership, quality trainings and performance indicators were observed. Community acceptance of young CMWs, professional jealousy, fear to lose CMWs, cooperation and demand of LHWs to work as CMWs were some of the major challenges faced. Nevertheless, developing a good policy is an important pillar to raise a strong building, whereas, making policies without good strategic plans can land up in wastage of many resources and creating new challenges to face. Moreover, to work under one umbrella with strong communication skills, sensitization workshops/trainings, performance based incentives for the workers are some of the salient features behind success of CBPs. Implementation of strategic communication of all stakeholders within the program, in between programs and with the community is the key to success CBPs.

### O3 Effect of age and gender on the clinicopathological features of bladder tumor

#### Javed Altaf, Muhammad Adeel Mahesar, Taimur Jatoi

##### Department of Urology, Liaquat University of Medical and Health Sciences, Jamshoro, Pakistan

*European Journal of Medical Research* 2017, **22(Suppl 1):**O3

The objective of this study was to observe the effect of age and gender on the clinic pathological features of bladder tumors in patients admitted at Liquate University Hospital Hyderabad, Sindh, Pakistan. This study was conducted for a period of 6 months at the Department of Urology, LUMHS. A total of 95 patients with bladder tumor were enrolled in this study. The bladder specimens of all the patients were sent for histopathological examination to find out the nature and grade of the tumour. Patient’s history and histopathological characteristics were recorded on a prescribed proforma. A total of 95 cases were enrolled in this study. Mean ± SD duration of disease was 2.1 ± 1.3 years. Out of 95 patients male to female ratio was 5.7:1. The average age of the patients was 60.04 ± 10.3 years. Most of the patients (n = 57) were between 50 and 59 years. Painless macroscopic hematuria was found in 61 males and in 8 females. Dysuria was found in 31 males and in 5 females. Urine urgency was present in 19 males and in 2 females. Majority of cases with painless macroscopic hematuria were between 50 and 59 years, Majority (47) of cases with dysuria had age between 50 and 59 years, majority of cases with urine urgency had age between 50 and 59 years, 16 (28.1%), but difference is insignificant (p = 0.2). In this study painless macroscopic hematuria and histological sub-type transitional-cell carcinoma was dominant with significant male preponderance among patients. Adult aged patients have low-grade disease.

### O4 Innovative health poems rural (desert) children

#### Jewat Sunder, Satti Jewat, Aziz Memon

##### Sindh United (n) Developmental Educational Rural Society, Rotary Club Khipro, Sindh, Pakistan

*European Journal of Medical Research* 2017, **22(Suppl 1):**O4

Polio is still crippling disease in Pakistan therefore we have need of strengthening of routine immunization as well as eradication of polio, it must be our first priority, by engage the community as well as school children by innovative activities to aware them about the importance of immunizations and eradication of polio. Our aim to eradicate polio free world, and object is educate, aware children about importance of polio drops. Descriptive study/with innovative health and analysis of data by asking questions and answers about immunization and polio and its importance. Sample size: 500 hundred children the results were: What do you mean by routine immunization? Answers were: 221 (44.2%) What is polio? Answers were: 353 (70.6%) What happen in polio? Answers were: 293 (58.6%) How many drops given? Answers were: 343 (68.6%) At what age groups taken polio drops? Answers were: 281 (56.2%) Innovative activities like health poems to develop a atmosphere of education by which children learn with interest, that interest will bring change and we have need of change for the saving lives of our beloved future for our beloved country as polio free.

### O5 Diagnostic evaluation of intrauterine fetal death with special reference of grey scale sonography at AKMCC Hyderabad

#### Hinna Feroz^1^, Roomi Aijaz^2^

##### ^1^Department of Radiology, AKMCC, Hyderabad, Pakistan; ^2^Department of Gynae LUMHS, Jamshoro, Pakistan

*European Journal of Medical Research* 2017, **22(Suppl 1):**O5

To evaluate causes of Intrauterine deaths with grey scale ultrasound with special reference from patient of Radiology Department AKMCC Hyderabad. Observational hospital based study was conducted at AKMCC on patient from 2011 up to August 2016 145 patients were included age 21–40 The primipara 44 multipara 101 booked cases of 35 have singleton pregnancy multipara 110 taking regular visit at ANC clinics. Average 96 with majority cases were gestational age 30–36 weeks. Patients assessed having no signs of viability at ANC visit were 100 cases incidentally diagnosed cases were 45. All patients were taken verbal consent. During study 145 total number patients were assessed for diagnosis of different causes of IUD with US. IUGR was the most common cause 45, oligohydramnios 26, placental abruption in 25, congenital anomalies were in 15, placental previa in 19 due to twin pregnancy one of fetal demise were 12 of total causes among IUD during given period. IUGR placental abruption congenital anomalies were assessed most prevalent causes of IUD. Advanced maternal age multigravidity was with increased risk of intra-uterine fetal death. Incidence of APH, Pre-eclampsia, eclampsia, diabetes and congenital anomalies showed lack of antenatal care Antenatal care with high risk patients and serial sonography at primary health care referral for Doppler, anomaly scan, nuchal translucency measurement by far the most important step in prevention of IUD and early diagnosis for the causes.

### O6 Runn of Kach earthquake in Tharparkar: its environment and mental health problems

#### Kolachi Hussain Bux

##### Isra University, Hyderabad, Pakistan

###### **Correspondence: Kolachi Hussain Bux**

*European Journal of Medical Research* 2017, **22(Suppl 1):**O6

2005 Pakistan suffered major earthquake in Kashmir and this year in October Gilgit and Northern areas. The deaths and public health problem are of similar nature but injuries and death in Tharparkar earthquake were comparatively very low. However the intensity on rector scale remained in similar ranges. We are sharing experiences and public health issues with Pakistani community after 14 years still people are remembering this painful experience. 2005 Pakistan suffered major earthquake in Kashmir and this year in October Gilgit and Northern areas. The deaths and public health problem are of similar nature but injuries and death in Tharparkar earthquake were comparatively very low. However the intensity on rector scale remained in similar ranges. We are sharing experiences and public health issues with Pakistani community after 14 years still people are remembering this painful experience.

**Materials and methods:** The study was conducted immediately after the earthquake through a Performa filled by medics and para-medics in total 220 questionnaires were filled of various health facilities under government of Sindh, who treated he earthquake survivors and physically suffered from earthquake tremors. The study was conducted from 26th January to 31st December 2001.

**Keywords:** Earthquake, Tharparkar, Environment.

### O7 Potential protective effect of salicylic acid on renal parenchyma damage by gentamicin in young albino rats

#### Muhammad Imran Rathore^1^, Samreen Memon^2^, Pushpa Goswami^3^

##### ^1^Department of Anatomy Muhammad Medical College, Mirpur Khas, Pakistan; ^2^Department of Anatomy LUM&HS, Jamshoro, Pakistan; ^3^Department of Anatomy LUM&HS, Jamshoro, Pakistan

*European Journal of Medical Research* 2017, **22(Suppl 1):**O7

Determine the preventive role of salicylic acid on renal parenchyma after given of gentamicin in young albino rats. For this experimental study, 30 young albino rats were taken. They were divided into three groups A, B and C. The animals in group-A given normal saline 6 ml/kg/day intraperitoneal for 2 weeks. Group-B received gentamicin 20 mg/kg/day intraperitoneal for 2 weeks and group-C receives gentamicin 20 mg/kg/day intraperitoneal with salicylic acid 20 mg/kg/day orally for 2 weeks. On day 15 all animals were sacrificed with deep ether anesthesia. Their kidneys were removed, fixed in 10% formalin. Representative blocks were taken and embedded in liquid paraffin. For routine histological examination 5 µm thick section cut by microtome and stained with H&E, PAS and silver methenamine. Renal histology was done under light microscope to see the proximal and distal tubular diameter and count. No significant (p > 0.05) changes were observed in the histopathology of kidney tissues of the groups A and C rats. In group B the Histopathology of Kidney is significantly (p < 0.001) affected. It may be concluded that gentamicin produces changes in kidney, which may be attributed to ischaemia resulting in tubular necrosis in young albino rats simultaneous administration of salicylic acid partially protect the morphological and histological changes induced by gentamicin.

**Keywords:** Gentamicin, Salicylic acid, Young albino rats, Kidneys.

### O8 Increased E/E′ ratio is a predictor of adverse outcomes after acute myocardial infarction

#### Jaweria Samejo

##### Department of Cardiology LUHHS, Jamshoro, Pakistan

###### **Correspondence:** Jaweria Samejo

*European Journal of Medical Research* 2017, **22(Suppl 1):**O8

Patients with acute MI are usually at high risk of developing complications i.e. arrhythmia, heart failure, recurrent attacks and death. Tissue Doppler imaging much more sensitive than 2D echocardiography for detecting LV systolic and diastolic functions abnormalities. Early diastolic velocity (Ea or E′) of the mitral anulus measured with TDI is a good indicator of LV myocardial relaxation Although E′ is reduced in patients with impaired relaxation and is affected less by preload than mitral inflow (E), thus the ratio E/E′ increases as PCWP increases. It is expected that a high E/E′ predicts a poor outcome. Seventy Patients of acute ST-segment elevation MI were included by purposive sampling. Echocardiography examination included both 2D as well as Doppler imaging. 52 in group 1 and 18 in group 2, with mean ± SD in group-I, hospital stay (days) was 3.7 ± 1.01 days, heart failure (any class) 1.88 ± 0.32, Arrhythmias 1.83 ± 0.38, Killip class-I = 4, Killip class-II = 1, Killip class-IV = 1 and 2.00 ± 0.00 death. On the other hand, in group II, it was found that hospital stay (days) was 5.1 ± 1.6 days, heart failure (any class) 1.55 ± 0.51, Arrhythmias 1.9 ± 0.26, Killip class-I = 2, Killip class-II = 3, Killip class-IV = 2 and 1.94 ± 0.23 death. Hospital stay, heart failure including killip class II, IV were statistically significant between the two groups but arrhythmias and mortality which were statistically non-significant. Above findings suggestive of that left ventricular filling pressure can be assessed by tissue Doppler echocardiography.

### O9 Acute upper gastrointestinal bleed patients requiring endoscopic intervention; assessment by Glasgow-Blatchford bleeding score

#### Mona Humaira, Kashif Zakria, Razia Hanif Ghani, Hanif Ghani, Shoaib Ansari

##### Department of Medicine LUMHS, Jamshoro, Pakistan

*European Journal of Medical Research* 2017, **22(Suppl 1):**O9

Upper gastrointestinal bleed is one of the most commonly encountered life-threatening complication of gastrointestinal disease. This study was conducted to assess the need of endoscopic intervention in patients with acute UGIB. This cross sectional descriptive study was conducted at Liaquat University Hospital, Jamshoro/Hyderabad from 05-January-2015 to 04-July-2015. Patients aged 18–65 years, of either gender presented with UGIB. In the form of hematemesis or melena of less than 24 h duration were admitted and evaluated for need of endoscopic intervention through Glasgow-Blatchford bleeding score. Total 113 patients were selected (82 males and 31 females) evaluated for need of endoscopic intervention in subjects with upper GI bleeding. Seventy-five (66.4%) patients had ≥ 02 GBS with male predominance (59 males). 49 patients were found to have single high risk component of GBS while 26 had multiple components of GBS. The mean age ± SD was 42.63 ± 7.75. The age in relation to gender, duration and endoscopic intervention was observed to be statistically significant (p > 0.01, p = 0.01 and p < 0.01) whereas the gender in relation to endoscopic intervention was also identified to be statistically significant (p = 0.04) respectively. In present study, 66.4% patients, predominantly male had GBS ≥ 2 underwent for endoscopic intervention. Clinical and lab based Glasgow Blatchford score is a very useful assessment tool for determining the need of endoscopic intervention in patients with acute upper gastrointestinal bleeding.

**Keywords:** Upper gastrointestinal bleeding, Endoscopy, Glasgow-Blatchford bleeding score.

### O10 Implementing need based MBBS curriculum

#### Muhammad Akbar Nizamani, Jan Muhammad Memon

##### Indus Medical College, Tando Muhammad Khan, Pakistan

*European Journal of Medical Research* 2017, **22(Suppl 1):**O10

Pakistan has poor indicators like high maternal and perinatal mortality rate, high infant and neonatal mortality rate, high rate of infectious diseases, poor immunization coverage, non optimum breast feeding, high ratio of malnutrition and high rate of death and disability due to major non communicable problems like metabolic syndromes. These problems are not adequately addressed in standard text books. Evidence of general practice in Pakistan has shown that medical graduates are ill equipped to serve the community with evidence based practices. Evidence has shown that curriculum based on community needs if implemented result in improvement of indicators. Unfortunately process of revisiting and implementing the curricular changes in Pakistan are very slow. At Indus Medical College a road map was selected by team work to start faculty development program to assess community needs and their place in present day curriculum. It was observed that students response to modular based curriculum was very positive. Early exposure of students to clinical problems increased their interest in better understanding of basic medical sciences. Faculty motivation and faculty time were identified as two major hurdles. This is however is a continuation process and impact of changes needs to be measured by an evidence based evaluation process. Revisiting MBBS curriculum and making it meaningful to respond to the needs of community is critically important. Implementing modular curriculum as per PMDC recommendations is need of the hour but will require an indigenous exercise by each degree awarding institution.

### O11 Results of health system strengthening for diabetic retinopathy and childhood blindness in District Hyderabad, Pakistan

#### Khalid Iqbal Talpur

##### **Correspondence:** Khalid Iqbal Talpur

*European Journal of Medical Research* 2017, **22(Suppl 1):**O11

###### Sindh Institute of Ophthalmology and Visual Sciences (SIOVS), Hyderabad, Pakistan

To present the results of Health System Strengthening Project for unknown diabetics, diabetic retinopathy (DR) and childhood blindness (CB) in District Hyderabad, Pakistan. Prospective data collection from January 2014 to December 2015 of screening for unknown diabetics, DR and CB in Hyderabad District. Lady health workers (LHWs) were trained to identify high risk (potential) patients for diabetes and childhood eye conditions and undertake preliminary screening and refer patients to basic health units (BHUs), where they were further assessed by Medical Officers (MOs) with the support of qualified optometrist, after which the patients were referred to Sindh Institute of Ophthalmic and Visual Sciences (SIOVS), @ Eye Hospital Hyderabad, Pakistan for management. A total of 995,244 population was covered in this screening program during which 2622 children (< 15 years) were screened for CB while 16,760 adult patients (> 15 years) were screened for diabetes. Random blood glucose level of 3075 patients was above 140 mg/dl. Out of these patients 17% patients were diagnosed with DR. Refractive error (42%) was the most common cause of childhood visual impairment. The diabetic screening program detected a high prevalence (17%) of retinopathy in diabetic patients living in Hyderabad District. The commonest cause of childhood visual impairment was refractive error (42%), which was successfully managed due to timely diagnosis. A large number of patients benefited from this screening program.

**Keywords:** Vision screening, Diabetes, Diabetic retinopathy, Childhood blindness, Community health.

### O12 Complexities of medical research in developing countries: standard of care: responsibilities of ethics committees

#### Inayat Ullah Memon

##### Indus Medical College, TM Khan, Hyderabad, Pakistan

###### **Correspondence:** Inayat Ullah Memon

*European Journal of Medical Research* 2017, **22(Suppl 1):**O12

Recognition of human rights particularly those of vulnerable and emphasis on avoidance of exploitation of weaker by stronger, have broadened the scope of bioethics in the field of research involving human subjects. Economic disparities between various nations and their differential health-care services, coupled with increasing research in medical sciences involving human subjects, particularly collaboration with for-profit organizations have opened new avenues of ethical debates including the issue of *standard of care in research in developing countries*. Emergence of newer infectious diseases and resurgence of older ones in recent past has prompted developed countries to undertake studies in under-developed areas. But it has generated complex and multiple ethical dilemmas. Ethical demands are that enrollees of research in developing countries not only be judiciously remunerated but outcomes of the studies be directly beneficial and affordable to them along with provision of parallel benefits. Focal point of debate among various stakeholders concerned with *standard of care in research involving human subjects in developing nations* is about obligation to provide universally best available care to participants. While some authors have put forward alternate standards while others relax the requirements of best standard of care under certain circumstances, conditional to the approval of Ethics Committees. These proposals have increased the responsibilities of the ERCs and IRBs to formulate contextual guidelines and overburdened them with task to disentangle the issues where existing international guidelines are insufficient. This paper discusses various aspects of this ethical issue, probes the alternate models and analyses various possible solution.

### O13 Identify the effect of maternal employment on child health under age of 10 years among the population of Qasimabad Hyderabad. Descriptive cross sectional study

#### Khuda Bux Mangrio

##### Qasimabad, Hyderabad, Sindh, Pakistan

###### **Correspondence:** Khuda Bux Mangrio

*European Journal of Medical Research* 2017, **22(Suppl 1):**O13

Descriptive Cross Sectional study. The study was carried out at Qasimabad Hyderabad. All working mothers with children of age < 10 years of either gender were the study population. The data was collected on a structured proforma and detailed information related to child co morbid illness due to employment was taken and Chi square test was applied. The mean maternal age was 37.6 years out of 290 respondents. The results of the present study showed 78.96% children were health affected due to maternal employment. Mostly 46.55% of children were GIT problems, respiratory problems were seen in 37.93% children where as anxiety was present in 12.06%. In the present study, there was significant relationship of maternal employment on child health such as anxiety, GIT problems and respiratory problems (p value 0.001). The results of this study indicate that maternal employment is an important determinant of a child’s risk of experiencing an adverse health event.

**Keywords:** Maternal employment, Child health, Age.

### O14 Reconstruction of scalp and forehead defects; a series of 76 cases

#### Shahzad Shaikh, Mahesh Kumar, Yasir Arafat, Naveera Fatima

##### Department of Plastic Surgery, LUMHS, Jamshoro, Pakistan

*European Journal of Medical Research* 2017, **22(Suppl 1):**O14

Reconstructive surgeries of scalp and forehead defects is a difficult task for which variety of techniques have been developed and used over the history in order to provide maximum surgical and cosmetic results. This study was planned to identify an appropriate reconstructive scheme. This prospective case series of 76 scalp and forehead defects was conducted in the Department of Plastic & Reconstructive Surgery, Liaquat University Hospital, Jamshoro, from March, 2012 to February 2016. 7 different surgical techniques were applied on 76 patients for reconstruction of scalp and forehead defects. These techniques included rotation flap, skin grafting, outer table drilling and primary closure etc. Variables estimated were etiology of scalp and forehead defects, reconstructive techniques and outcomes of the techniques. Among 76 patients, there were 74% male and 26% female. Most common cause of scalp and forehead defects was direct trauma (31.57%), followed by electric injury (21.05%) and tumor (18.42%). Rotation flaps and skin grafting were the most used techniques and the complications were occurred only 18.42% of the total patients. Scalp and forehead defects should effectively be managed by applying durable coverage after adequate debridement, proper wound drainage and by preserving blood supply. Rotation flaps and skin grafts stay on being spine to reconstructive techniques for scalp and forehead defects in most instances.

**Keywords:** Scalp and forehead defects, Rotation flaps and skin grafting, Reconstructive surgeries.

### O15 Correlation of atrial fibrillation to left atrial size

#### Mariya Qazi, Syed Fasih Ahmed Hashmi, Muhammad Ali Bohyo

##### Department of Cardiology, LUMHS, Jamshoro, Pakistan

*European Journal of Medical Research* 2017, **22(Suppl 1):**O15

Atrial fibrillation is the most common arrhythmia and has a strong correlation is observed in cases of rheumatic mitral valve heart diseases. To correlation of atrial fibrillation with left atrial size in patients of Rheumatic mitral valve heart diseases Prospective observation descriptive study. 120 Cases admitted in Cardiology Department LUMHS, Hyderabad with detailed clinical history and examination suspected rheumatic mitral valve cardiac disease were registered by non probability purpose technique during a period from April 2015 to March 2016. These registered patients further evaluated. ECG was done for atrial fibrillation and Echocardiography was performed by using an advanced technology ultra mark 6 ultrasound Toshiba to calculate left atrial size. Mean age of cases in this study was 37.83 ± 16.39 years. Females were found in majority 80/(33.3%) as compare to male 40/(66.7%). Breathlessness and palpitation were found more frequent exhibiting complains in 92/(76.6%) and 54/(45.0%) cases. In all cases of this study history of rheumatic fever was found. In this study strong correlation was found between atrial fibrillation and left a trial size p value = 0.001. Left atrial size independently correlates with the development of atrial fibrillation. The incidence of atrial fibrillation is significant in rheumatic heart diseases. It is higher in females. More such studies are required to evaluate the more satisfactory correlation.

**Keywords:** Atrial fibrillation, Rheumatic mitral valve heart diseases, Echocardiography, Left atrium.

### O16 Intraumbilical infusion of syntocinon as an efficacious route for prevention of complications of 3rd stage of labour:*“ROUTE does MATTER”*

#### Roomi Aijaz, Seema Bibi, Raheel Sikundar, Hinna Feroz

##### Department of Gynae and Obs, Liaquat University Hospital, Sindh, Pakistan

*European Journal of Medical Research* 2017, **22(Suppl 1):**O16

To determine the efficacy of intraumbilical syntocinon by (papingas technique) in comparison of intravenous syntocinon for managing the 3rd stage of labour. Randomized controlled trial (interventional study), Department of Gynae and Obs unit 1 LUMHS hospital Hyderabad from March 2007 to March 2008. 100 parturient women with low risk singleton term pregnancy were recruited in the study. 50 pts were recruited in the study and control group after randomization. Three primary outcome measures were kept amount of blood loss and duration in third stage of labour. As the 3rd stage complication incidence of retained placenta, post partum heamorrhage or retained clots were observed as complications. Women in study group who received intra umbilical syntocinon were measured less blood loss comparatively with control (p = 0.005). Duration of third stage of labour was also significantly low in study group compared to control group 3.7 (1.5) min vs. 7 (7.25) min (p = 0.0005). Regarding complications no significant difference was observed between groups with (p = 0.27). In this study intraumbilical vein syntocinon by (papingas technique) is proved a efficacious route in managing the complications of 3rd stage of labour to reduce the maternal morbidity and mortality.

### O17 The novel heterozygous Asp217Asn CYP1B1 gene mutations causes severe primary open angle glaucoma (POAG) in large Pakistani family

#### Yaqoob Shahani, Samreen Memon, Ali Muhammad Warya, Umbreen Bano, Pashmina Sheikh, Samina Gul

##### Department of Anatomy, Liaquat university of Medical & Health Sciences, Jamshoro, Pakistan

*European Journal of Medical Research* 2017, **22(Suppl 1):**O17

Glaucoma, a group of complex disorders is the major cause of irreversible blindness throughout the globe. Primary open-angle glaucoma (POAG), the most common type is characterized by optic disc damage, visual field defects and elevated or normal intraocular pressure (IOP), with no evidence of angle closure on Gonioscopy. Although the exact cause of POAG is still not known, but genetic mutations have roles in the etiology of POAG. This study was conducted to detect the mutations of CYP1B1 gene in familial cases of Primary Open Angle Glaucoma in the population of Sindh province of Pakistan. This study was conducted at Liaquat University of Medical & Health Sciences, after ethical approval from Departmental Ethical review committee and informed consent from patients. Total 25 families of POAG with more than one affected member were enrolled; disease was confirmed with ophthalmological examinations. Blood samples were collected from enrolled families. Genomic DNA was extracted from whole blood for genotyping and sequencing. Predicted role of novel variants of CYP1B1 was assessed by bioinformatics tools. The role of CYP1B1 gene in the pathogenesis of POAG was identified in one family. CYP1B1 gene sequencing revealed, A novel heterozygous transition c.650 G>A, substituting Aspartic acid into Asparagine at codon 217 (p. Asp217Asn) of CYP1B1. This novel mutation is detected for the first time in any Pakistani family with POAG and is detected in all the affected family members of this large family.

### O18 Effects of supplemented chromium on maternal health

#### Umbreen Bano, Samreen Memon, Yaqoob Shahani, Pashmina Sheikh, Samina Gul

##### Department of Anatomy, Liaquat University of Medical & Health Sciences, Jamshoro, Pakistan

*European Journal of Medical Research* 2017, **22(Suppl 1):**O18

Pregnant women are prone to certain micronutrient deficiencies, due to increase metabolic demands which results in unfavourable maternal and fetal health effects. This study was aimed to detect the possible effects of diet rich in chromium during intrauterine life on maternal health. Adult female mice divided in two groups of 12 animals each, were housed in standard environment. Control group was fed ad libitum. Second group was given chromium rich diet (0.51 mg/kg diet). The animals were kept on these diet regimens for 4 weeks. Plasma levels of these trace elements were determined at the end of 4 weeks of feeding regimen. After ensuring the levels, the animals were allowed matting. The pregnant mice were fed on same diet throughout pregnancy. Daily food intake and weekly body weight was monitored throughout the duration of study, and the effects of this trace element was observed on pregnant mice. At the end of the study animals were sacrificed for microscopic examination. Gross examination revealed significant increase in weight of pregnant mice supplemented with chromium as compared to control. Microscopic examination showed lymphocytic infiltration, necrosis and infarction in the cardiomyocytes, cholestasis (intracellular, intraductular) in hepatocytes, interstitial mononuclear lymphocytic infiltration in glomeruli, and lymphocytic infiltration around islet of Langerhans in pancreas. This animal study showed that micronutrients if used in excess during pregnancy might be harmful to the mother and should be taken as advised. Further animal studies are needed to examine the effects of different micronutrients on pregnancy.

### O19 Outcome of fasciocutaneous flaps in exposed bones of leg

#### Naveera Rafique

##### Plastic Surgery Department, Liaquat University of Medical & Sciences, Jamshoro, Pakistan

###### **Correspondence:** Naveera Rafique

*European Journal of Medical Research* 2017, **22(Suppl 1):**O19

The aim of present study is to document the etiology of the leg defect and to highlight the role of different reconstructive techniques for the management of exposed leg bones. Department of Plastic and Reconstructive Surgery L.U.M.H.S, Jamshoro Jan 2014 to Dec 2015. The patients of both sex and age, presenting with simple or compound defect of the leg from any cause were included in the study. During period of the study 100 patients having simple or compound defect of leg were managed by different reconstructive technique. Results will be shown in the presentation. We concluded that exposed bone at any site of tibia and foot can be covered by antegrade or retrograde fasciocutaneous flap.

### O20 Possible preventive role of glutathion against oxaliplatin induced neurotoxicity, behavioral and morphological appearance of an adult albino mice

#### Pashmina Shaikh, Samreen Memon, Samina Memon, Umbreen Bano, Yaqoob Shahani

##### Department of Anatomy, Liaquat University of Medical & Sciences, Jamshoro, Pakistan

*European Journal of Medical Research* 2017, **22(Suppl 1):**O20

Oxaliplatin (Ox), is a drug of choice in colorectal cancers and other tumors. Adverse effects include peripheral Neuropathies, neurotoxicity, ototoxicity, weight loss and GIT symptoms. The antioxidants have been used to reduce the toxicities produced by chemotherapeutic drugs. This study is aimed to assess the preventive role of glutathione on Ox induced neurotoxicity, morphological appearance and behavior of adult albino mice. This study was conducted at Anatomy Department LUMHS and animal house Sindh Agriculture University TandoJam for the period of 1 year. Adult mice of 32–35 g were divided into 3 groups and were given divided doses for 8 weeks. GROUP A (control), GROUP B: OX treated mice (0.02 mg/sc), GROUP C: mice given OX (0.02 mg/sc with glutathione 500 mg/PO). Gross examination and behavioral tests were observed during the whole duration. The weight of the group B significantly decreased over the entire study period of study as compared to group A (p < 0.05). Other gross changes include loss of skin hair, paw edema or paralysis, mental orientation, object recognition, noise stimulation in group B exhibit significant difference to control p < 0.05. Behavioral tests such as heat and cold stimulation, the time to move away from the stimulus significantly increased in group B p < 0.05. However, group C animals were comparable to group A in gross appearance and behavior. This study showed the toxic side effects of OX on gross morphological & behavioral of adult albino mice. Addition of glutathion reversed the toxicity produced by OX.

### O21 Treatment of melasma with microdermabrasion and combination of topical skin care regimen 22% ascorbic acid (AA), 19% glycerin (G) and 5% borage vegetable oil (BVO)

#### Sobia Wali Muhammad

##### Department of Dermatology, LUMHS, Jamshoro, Pakistan

###### **Correspondence:** Sobia Wali Muhammad

*European Journal of Medical Research* 2017, **22(Suppl 1):**O21

Melasma is common disfiguring and stressful condition which is refractory to treatment. Mostly, long term remissions are difficult to achieve. The purpose of our study is to evaluate the safety and efficacy of microdermabrasion and combination of topical skin care regimen 22% ascorbic acid (AA), 19% glycerin (G) and 5% borage vegetable oil (BVO) in the treatment of refractory melasma. In this study, eighteen female patients who fulfilled the inclusion criteria were selected. Skin type was identified with the help of wood’s lamp. All participants underwent microdermabrasion twice monthly for 3 months with combined treatment of skin care regimen. Monthly follow up assessment was done during treatment. According to our study result, this painless combined treatment was successful in all participants. Most of the patients results in fifty percent of clearance in first month of therapy. Two patients noted mild exacerbation of melasma with sun exposure however; this was resolved with topical skin care regimen. Due to dramatic improvement of melasma, an excellent compliance was observed in patients. Therefore, it can be concluded that in Asian population combined treatment of microdermabrasion with topical regimen were found safe and effective in refractory melasma.

### O22 Hypospadias: a comparative study between Snod Grass and Braca’s procedure

#### Yasir Arfat Memon, MaheshKumar, Shehzad Sheikh

##### Liaquat University of Medical & Sciences, Jamshoro, Pakistan

*European Journal of Medical Research* 2017, **22(Suppl 1):**O22

To evaluate the outcome of Snod grass and Brac’s procedure in LUH Jamshoro. Two years from January 2014 to December 15. Observational and comparative study. Total number of patients ar 50, 25 patients selected for Snod Grass and 25 patients selected for Braca’s procedure. According to our study Braca’s procedure have superior results then Snod grass.

### O23 Relationship between *Helicobacter pylori* (*H. pylori*) infection, eradication therapy and body mass index (BMI)

#### Muhammad Khalid Shaikh

##### LUMHS, Jamshoro, Pakistan

###### **Correspondence:** Muhammad Khalid Shaikh

*European Journal of Medical Research* 2017, **22(Suppl 1):**O23

**Background:** One of the most common etiological factors associated with chronic gastritis, duodenal ulcer and gastric carcinoma is *H. pylori*. This study was conducted to evaluate the effects of *H. pylori* eradication therapy on BMI of the patients.

**Methods:** This cross sectional study was carried out at Department of Medicine, Liaquat University Hospital, Jamshoro, Pakistan on 137 patients diagnosed with *H. pylori* infection. BMI and weight of the patients was measured and symptoms were observed before and after 3 months of completion of therapy at follow up. The assessment of symptoms was performed by applying Mann–Whitney U test of significance.

**Results:** After eradication therapy, average BMI was increased from pretreatment values of 23.24 ± 2.1 to 26.4 ± 3.8 kg/m^2^ (p = 0.028). Also a significant different in body weight was observed from pre-treatment mean body weight of 61.7 ± 12.3 to 62.5 ± 11.2 kg (p = 0.011). Mean score of symptoms such as dyspepsia decreased from 2.65 to 1.02 (p = 0.024). Other symptoms such as epigastric pain reduced from 2.73 to 1.14 (p = 0.041), upper abdomen fullness reduced from 2.49 to 0.35 (p = 0.021). A decrease in heart burn and loss of appetite was also observed {3.46 vs 0.92 (p = 0.003)} and {2.89 vs 0.58 (p = 0.011)} respectively after eradication of *H. pylori*.

**Conclusions:** Eradication therapy is positively associated with increased BMI and body weight after completion.

**Keywords:** Eradication therapy, *Helicobacter pylori*, BMI.

### O24 Red meat consumption and coronary artery disease

#### Gulshad Wagan, Samreen Memon

##### Department of Anatomy, Liaquat University of Medical & Health Sciences, Jamshoro, Pakistan

*European Journal of Medical Research* 2017, **22(Suppl 1):**O24

Red meat intake is associated with stroke, chronic heart disease and metabolic syndrome. However white meat is considered a healthy substitute to the red meat with same health benefits. This study was aimed to examine the effects of red meat and white meat on the coronary arteries of adult albino male mice. This experimental study was carried out at Anatomy Department Liaquat University of Medical and Health Science (LUMHS) Jamshoro in Collaboration with Animal House of Sindh Agriculture University Tando Jam from August 2015 to January 2016. 60 adult male mouse average weight of 20–25 g were used. The animals were divided in four groups; group A (n = 15) control mice were fed on normal diet, Group B (n = 15) given white meat, group C (n = 15) were given red meat and Group D (n = 15) were fed on white and red meat both for 6 months. At the end of study period blood sample was collected for cholesterol levels and animals were sacrificed by cervical dislocation and small pieces of coronary arteries were dissected and collected in 10% formalin. Coronary artery sections were processed for microscopic examination. This study shows that consumption of red meat increased the levels of blood cholesterol as compared to control and animals fed on white meat. Histological features of coronary arteries showed thickness of tunica intima as compared to other groups (p < 0.05). Consumption of red meat is related to high levels of blood cholesterol and increased thickness of coronary walls.

### O25 Increasing rate of caesarean section due to non-reassuring CTG

#### Pushpa Chetan Das, Sana Zahiruddin, Neeta Sham, Nigar Jabeen

##### Liaquat University Hospital, Hyderabad, Pakistan

*European Journal of Medical Research* 2017, **22(Suppl 1):**O25

To evaluate increasing rate of caesarean section due to non-reassuring CTG. Cardiotocography (CTG) is the graphic presentation of fetal heart activity and the uterine contraction to detect the fetal hypoxia. It is the most commonly used test for antepartum and intrapartum fetal surveillance in the majority hospitals of developed countries. This technology was first developed in 1950 and became commercially available in 1960. The goal of antepartum fetal surveillance is to predict, diagnose and timely intervene the pregnancies those are complicated with fetal asphyxia and might lead to fetal and newborn morbidity and death. IUGR (intrauterine growth restriction) fetus are at risk of fetal distress and this can be detected by using CTG. CTG examination consist of tocogram and ultrasound transducer, tocogram monitor uterine contraction while ultrasound transducer record fetal heart rat. Fetal academia metabolic (95.5%) mixed (95%) and respiratory (100%) can be significantly detected by fetal heart rate monitoring. The pathologic finding of CTG during labor may increase the caesarean rate. According to WHO recommendations the Caesarian section rate is 15%. The rate of caesarean section due to fetal distress monitored by CTG from 9.6 to 19% as given in different studies. CTG is a useful and indispensable adjunct to monitor the condition of endangered fetus. However, there is a need to develop a standardized and unambiguous definition of FHR tracing to reduce the incidence of false positive findings that may result in increased incidence of unnecessary intervention particularly caesarean section.

### O26 Liver fibrosis stage in viral hepatitis patients following recurrence after conventional treatment

#### Sanam Maree, Binafsha Manzoor Syed, Bikha Ram Derajani

##### Shearwave Elastography Unit, Medical Research Centre, Liaquat University of Medical & Health Sciences, Jamshoro, Pakistan

*European Journal of Medical Research* 2017, **22(Suppl 1):**O26

Viral hepatitis is a major risk factor for development of liver fibrosis. This study aims to assess stage of liver fibrosis in patients with Hepatitis B and C after recurrence. All patients with a confirmed diagnosis of recurrent viral hepatitis B and C came to the shearwave elastography unit during a period of 30 months (i-e April 2014–October 2016) were included. All patients recurrence after conventional therapy. Their liver fibrosis was assessed by using Supersonic imagine Aixplorer, shearwave elastography system. The liver fibrosis stage was defined according to metavir scoring system. A total of 3347 patients had confirmed viral hepatitis, 1304 had hepatitis C and 550 had hepatitis B positive after conventional treatment. Out of these patients 9.3% had normal liver, 28.7% had fibrosis stage 1, 22.4% stage 2, 16.8% had stage 3 and 22.8% presented with stage 4 liver fibrosis. The patients with recurrence of viral hepatitis had higher chances of presenting with high liver fibrosis and develop cirrhosis. Therefore a close check on liver fibrosis stage is utmost important.

### O27 Outcome of sublay mesh repair in ventral hernia

#### Altaf Talpur

##### Department of Surgery, Liaquat University of Medical & Health Sciences, Jamshoro, Pakistan

###### **Correspondence:** Altaf Talpur

*European Journal of Medical Research* 2017, **22(Suppl 1):**O27

Ventral Herniae are common surgical problem faced by General Surgeons worldwide. Sublay mesh repair is gaining popularity due to better results so far in the literature. This study aims at finding out the results of sublay mesh repair in our part of the world which has significant burden of this type of disease. Prospective study conducted from Jan 2014 to Aug 2015 at public and private sector Hospitals of Hyderabad and Karachi. It includes all patients of either sex with age more than 13 years having different types of ventral herniae. Patients of ventral hernia operated in emergency condition or in which mesh was not implanted due to any reason were excluded. Also patients who lost to follow up or unwilling to participate in study were excluded. During this period 59 patients of Ventral hernia were operated by sublay mesh technique. It includes 47 female & 12 male with mean age of 44.69 ± 13.974 years. Most common type of hernia was paraumbilical hernia in 37 (62.71%) patients followed by Incisional hernia in 09 (15.25%) patients. Mean BMI was 26.86 ± 5.575 kg/cm^2^. Nine (15.25%) patients had co morbid illness with diabetes mellitus in 03 (5.08%) patients. Mean operative time was 62.14 ± 15.558 min with average postoperative hospital stay of 58.08 ± 15.986 h. Postoperative complications were noticed in 06 (10.16%) patients. It includes seroma formation in 03 (5.08%) patients, superficial wound infection, mesh infection and recurrence in 1.69% patient each. Sublay mesh repair for ventral hernia is gaining popularity due to early recovery & less postoperative complications.

### O28 Neonatal admissions with sacrococcygeal teratoma at Liaquat University Hospital, Jamshoro, Pakistan

#### Pushpa Goswami^1^, Mahesh Kumar^2^, Samreen Memon^1^

##### ^1^Department of Anatomy, LUMHS, Jamshoro, Pakistan; ^2^Department of Plastic Surgery, LUMHS, Jamshoro, Pakistan

*European Journal of Medical Research* 2017, **22(Suppl 1):**O28

Sacrococcygeal teratoma (SCT) is a less common congenital tumour with paucity of literature in South Asian region. Early diagnosis and proper management can reduce the risk of long term morbidity and mortality. This study was conducted in the Pediatric Surgery Department, Liaquat University Hospital, Sindh Pakistan over a period of 5 years (2010–2015). During study period 10 patients with SCT enrolled through outpatient department and were included in this study. All patients underwent surgical excision after detailed history and physical examination. During study period total 10 patients were admitted and operated. Out of those 10 patients 06 were males, 04 were females with age ranging between 20 h and 08 months. All (09) were full term while only (01) was preterm baby. Four were delivered by normal vaginal delivery and six by cesarean section. Only 03 were diagnosed by ultrasound in antenatal period while 07 in postnatal period. SCT is tumor of neonates and infants which require surgical excision by team of experts including pediatric surgeon, neonatologist, neurosurgeon and anesthetic. The excision of tumor mass extending in nearby viscera and vascular tumor require expert skills with good anesthetic approach in young patients. Better surgical outcomes are possible provided surgery in proper time and be properly performed. This study reveals high male to female ratio, might be the fact that in third world countries less attention is given to females.

### O29 Relation of erectile dysfunction (ED) to mortality after coronary artery bypass grafting (CABG)

#### Sharjeel Abbas, Abdul GhaffarMemon, Adeel Abbas, Madiha Iqbal, WaseemRiaz, Mohsin Hussain

##### Liaquat University of Medical & Health Sciences, Jamshoro, Pakistan

*European Journal of Medical Research* 2017, **22(Suppl 1):**O29

Erectile dysfunction is long being linked with greater risk of cardiovascular events. The study aims to find out association of presence and severity of erectile dysfuction with postoperative mortality in patients undergoing CABG. Prospective Observational Study, with consecutive sampling. Out Patient Department of Cardiothoracic Surgery and Cardiology, LUMHS, Jamshoro/Hyderabad. From October 2015 to October 2016, thirty-Seven (37) men aged 45–60 years who underwent CABG were include were included. An international index of erectile function 5 (IIEF-5) was used as a questionnaire given to patients at follow-up visits on 1st, 6th and 12th months postoperatively. Eleven (11) patients were found to have erectile dysfunction, out of 11 patients only 5 cases completed the trial, whereas six cases were lost to follow up between 6th and 12th months post CABG. From the 5 patients who completed the study, all were asymptomatic at 1st and 6th month followups, while only two cases remained asymptomatic at 12th month followup. No mortality observed at 1st and 6th month followups, whereas four patients were dead (from those who were lost to followup) when relatives were contacted at telephone at 12th month. Those who completed the study, three patients were in NYHA Class 3. There is significant relationship between erectile dysfunction and mortality after coronary artery grafting.

### O30 Clinico hematological features in children and adults diagnosed as idiopathic thrombocytopenic purpura at diagnostic & research lab Hyderabad

#### Fahmeena Qadri, Ikram din Ujjan

##### Department of Pathology, LUMHS, Jamshoro, Pakistan

*European Journal of Medical Research* 2017, **22(Suppl 1):**O30

Idiopathic thrombocytopenic purpura (lTP) is also known as immune thrombocytopenic purpura generally defined as a platelet count of less than 100 × 10^9^/l and is the commonest cause of thrombocytopenia in childhood. It results from an immune mediated destruction of circulating platelets within the reticuloendothelial system, mainly in the spleen. This study was aimed to find the frequency of common clinical features of Idiopathic Thrombocytopenic Purpura (ITP) in children & adults presenting to diagnostic and research lab Hyderabad. A cross sectional descriptive study design was used and 50 patients presenting with bleeding through any orifice with diagnostic evidence of ITP were selected through non random convenient sampling. Common clinical features were noted along with hematological parameters. The cases were then managed according to standardized management criteria. A total of 347 patients were observed during February 2014 to 2016. In which 50 diagnosed as having ITP. Bruising (46%), Epistaxis (36%) and Petechiae (25%) were the most common clinical features. Platelet count was reduced in 38 out of 50 cases with median count of 135,000/mm^3^. Bruising, Epistaxis and Petechiae are the most common features of ITP.

## Poster presentations

### P1 Elevated hemoglobin-F levels in macrocytic anemia

#### Abdul Rehman Shaikh, Ikram Din Ujjan, Arshi Naz

##### Diagnostic and Research Lab, Hyderabad, Pakistan

*European Journal of Medical Research* 2017, **22(Suppl 1):**P1

Fetal hemoglobin (HbF) is the main hemoglobin component throughout fetal life and at birth, accounting for approximately 80% of total hemoglobin in newborns. After birth, HbF synthesis rapidly declines and HbF is gradually substituted by HbA in the peripheral blood, so that within the first 2 years of life, the characteristic hemoglobin phenotype of the adult with very low levels of HbF (less than 1%) is found. Macrocytic anemia describes an anemic state characterized by the presence of abnormally large RBCs in the peripheral blood. Macrocytosis due to vitamin B12 or folate deficiency is a direct result of ineffective or dysplastic erythropoiesis. To observe the hemoglobin pattern in macrocytic anemia patients, referred from various parts of Hyderabad city. 4 ml blood was drawn and CBC was performed on XN 1000 six part fully automatic analyzer. Slides were stained by Leishman stain. Hemoglobin electrophoresis was performed on HPLC Variant II by Bio Rad. The data was retrospectively analyzed and all the patients with MCV greater than 95 fL were short-listed. Out of 1612 subjects studied, 68 patients had an MCV greater than 95 fL, out of which 41 were males and 27 were females. Out of these 68 patients under study, 48 had a raised hemoglobin-F level according to age. This research indicates that there might be an association of macrocytosis with a raised hemoglobin-F level, which may be further detected on gene mutation analysis studies.

**Keywords:** Megaloblastic anemia, Hemoglobin F, Macrocytic anemia.

### P2 Epidemiology of skin disease in LUHMS Hospital OPD, health care seeking barriers and recommendations to overcome them

#### Abdullah Kamran Soomro, Doulat Bajaj, Shaista Shah

##### Liaquat University of Medical & Health Sciences, Jamshoro, Pakistan

*European Journal of Medical Research* 2017, **22(Suppl 1):**P2

About 10–15% of population from Sindh is suffering from skin diseases, and the proportion is rampantly on the rise, owing to factors related to the environment, hygiene, suffocation, cosmetic use, etc. It is a cross-sectional study on a total of 3747 patients presented in the Dermatology outpatient department of Liaquat Medical Hospital Hyderabad, spanning from June 01, 2014 to December 31, 2014. To find out the Epidemiological distribution of the disease among visiting patients; To determine the impediments & barriers in seeking the health care; and most important of all, to place recommendations for better health care provision, pertaining curative as well as preventive measures, making appropriate use of technology & resources at hand. The patients were of all age groups with a preponderance of females on males. The most common problem was scabies, followed by eczema, fungal infections, acne, bacterial infections, and a list of diseases in decreasing frequency. The recommendation section of the study shows the methodology to be adopted and the probable cost incurrences for the project. The proposal is pricelessly valuable in (a) establishing linkages between the community and the health care provision at the grass-root level, (b) improving better referral system AND (c) providing ample opportunity to collect value data for the purpose of research and scientific evidence collection.

### P3 Use of medical drugs and risk of aplastic anemia: a case control study

#### Muhammad Asif Syed^1^, Aneela Atta Ur Rahaman^2^, Tahir Sultan Shamsi^3^

##### ^1^Liaquat University of Medical and Health Sciences, Hyderabad, Pakistan; ^2^Department of Community Health Sciences, Liaquat University of Medical and Health Sciences, Jamshoro, Pakistan; ^3^Department of Clinical Hematology, National Institute of Blood, Disease Center and Bone Marrow Transplantation, Karachi, Pakistan

*European Journal of Medical Research* 2017, **22(Suppl 1):**P3

Aplastic anemia is strongly linked with use of numerous medical drugs in developed countries. Unregulated use of pharmaceutical drugs in low middle income countries has been suspected of causing large numbers of aplastic anemia cases but epidemiologic evidence is limited. The purpose of study was to determine the association of medical drugs with aplastic anemia. A hospital-based case–control study was conducted in Karachi, Sindh with 89 cases and 356 age and sex matched controls. Cases were patients of aplastic anemia confirmed through bone marrow biopsy. Exposures of drugs and other potential risk factor prior to diagnosis were collected by in-person interview. Odds ratios and their 95% confidence intervals were calculated by using univariate regression models. We observed an elevated odds ratio (OR) estimates for sulfonamide (OR 6.3: 95% CI 2.3–17; p = 0.00) and carbamazepine (OR, 3.4: 95% CI 1.6–11; p = 0.2). Among the NSAID only salicylates has an evidence of significant association (OR, 3.5: 95% CI 1.5–7.8; p = 0.1) with aplastic anemia. Individuals exposed to thiazide (OR, 3.4: 95% CI 1.0–11.6; p = 0.02), interferon (OR, 4.1: 95% CI 1.1–14.7; p = 0.01) and mebendazole (OR, 4.1: 95% CI 1.1–14.7; p = 0.01) had positive link with aplastic anemia. No associations was identified with benzodiazepines, antihistamines, oral contraceptives, and herbal preparations. This study provide evidence that use of some traditional drug like sulfonamide, carbamazepine, salicilates, thiazides and mebendazole related to aplastic anemia. Physicians should be attentive to the possibility of drug associated aplastic anemia.

**Keywords:** Drugs, Aplastic anemia, Risk factors, Case control study.

### P4 Protective role of vitamin E against cisplatin induced alterations in histology of kidneys and renal function tests

#### Sameena Gul, Samreen Memon, Pashmina Shaikh, Umbreen Bano, Yaqoob Shahani

##### Department of Anatomy, LUMHS, Jamshoro, Pakistan

*European Journal of Medical Research* 2017, **22(Suppl 1):**P4

Cisplatin was the 1st platinum based compound approved for the treatment of various organ cancers. It results in several toxicities but the renal toxicity is its chief side effect. Vitamin E (Tocopherol) is an antioxidant with promising results on prevention of toxicities due to chemotherapy. This study was designed to determine the protective role of vitamin E against Cisplatin induced alterations on the histology of the kidneys and the renal function tests of the adult mice. This experimental study was conducted at LUMHS and Animal house of Sindh Agriculture University Tando Jam for period of 1 year. Sample size was 60 adult mice with average weight of 30–50 g, randomly divided into three groups as Group A (Control), Group B (Cisplatin only), Group C (Cisplatin + Vitamin E). During and at the end of the study period, blood samples were taken for renal function tests. After completion of drug cycles, the mice were dissected & the kidneys were preserved for H&E staining. Histological features of Group B showed necrotic & acute tubular ischaemic changes while Group C showed normal histological features with some areas of focal lymphatic infiltration (p < 0.05). Blood urea nitrogen (BUN) & creatinine levels were increased in Group B as compare to Group C. Vitamin E was successful in reversing the effects of Cisplatin to a certain extent (p < 0.05). Addition of antioxidant (Vitamin E) dramatically reversed the toxic effects of cisplatin on kidneys in Group C.

**Keywords:** Cisplatin, Vitamin E, Renal toxicity, Histology, RFT.

### P5 Sustainable global health agenda and health policy and system research

#### Aijaz Qadir Patoli^1^, Nusrat Sehto^2^, Shaista Aijaz^3^

##### ^1^Sir C.J. Institute of Psychiatry, Hyderabad, Pakistan; ^2^Government Hospital, Qasimabad, Pakistan; ^3^Liaquat University Hospital, Hyderabad, Pakistan

*European Journal of Medical Research* 2017, **22(Suppl 1):**P5

Health is central to development. In the post industrial revolution 200 years of development; the world demography has now soared from 400 million to over 7 billion and urban population from 10% to over 50%. Sustainable development envisages economic development, social inclusiveness and environmental sustainability. Health Goal 3 of Sustainable Development Goals SDGs (2016–2030) places universal health coverage UHC at the center, aiming at equitable & accessible quality health with least financial burden & to be enforced in country contexts through priority-setting. This System perspective has led to emergence of new field of Health Policy and System Research HPSR focusing on relationships between Health Research & Health Systems & on broader Health Determinants. HSPR also integrates functioning of researchers, policy makers & communities to address the complex health challenges to find the sustainable solutions. “The Alliance for Health Policy and Systems Research” is a global health partnership of 350 plus governmental & UN agencies and academic & research institutions hosted by WHO that works with the institutions to ensure sustainability. Since 2007 Alliance began to establish Systemic Review Centers SRCs in LMICs, bridging gap between policy makers and researchers with objective to improve performance of health systems through evidence-based informed policies. Pakistan has readiness for global partnerships as larger population use internet and mobiles. There is promising potential in our professionals for Health Research but institutional capacities are limited. Our institutions should engage in global health partnerships to synchronize local health policies with the global health agenda.

### P6 Clinical significance of immature platelet fraction and CD4+/CD8+ ratio in patients with immune thrombocytopenic purpura

#### Aisha Arshad, Samina Naz Mukry, Madiha Saud, Iffat Shamim, Muhammad Nadeem, Tahir Shamsi

##### National Institute of Blood Diseases, Karachi, Pakistan

*European Journal of Medical Research* 2017, **22(Suppl 1):**P6

Immune thrombocytopenic purpura (ITP) is an autoimmune clinical syndrome in which decreased number of circulating platelets leads to epistaxis, bruises and bleeding. The T cells play an important role in the pathogenesis of ITP. A total of 50 ITP patients and 50 aged and sex matched healthy volunteers were included in this study as control. Informed consent was taken from all the patients. Complete blood count was performed on automated hemo-analyzer XN-1000 and morphological examination of blood was done in duplicate by two different microscopists. The CD4+/CD8+ ratio was determined by BD Tri-test CD4 FITC/CD8 PE/CD3 PerCP antibody cocktail using BD FACS Calibur flow-cytometer. The data was acquired and analyzed by using Multiset version 3.0. Out of total 50 patients, 21 (42%) were males and 29 (58%) were females. Platelet anisocytosis and decreased platelet count was observed. The mean platelet count was 28.2 × 103/Âμl and 328 × 103/μl for ITP patients and controls respectively. The most commonly observed symptoms were epistaxis and bruises with a frequency of 13–14%. The immature platelets fraction (IPF) was raised i.e. 18.14 in ITP patients whereas the CD4+:CD8+ ratio was also 0.05-fold higher in ITP with a mean value of 1.29 (0.39–3.99). A significantly strong positive correlation was observed between CD8+ Tc cells and CD4+:CD8+ ratio (r = 0.974; p < 0.05). In this study decreased platelet count and low CD4/CD8 ratio were observed. This decreased ratio can be used as a marker in evaluating the immune-suppressive drugs efficacy/response in ITP patients.

### P7 Better preservation of right ventricular function with retrograde cardioplegia

#### Ammar Hameed Khan^1^, Sharjeel Abbas^2^, Muhammad Muneeb^1^, Madiha Iqbal^1^

##### ^1^Punjab Institute of Cardiology, Lahore, Pakistan; ^2^Liaquat University of Medical & Health Sciences, Jamshoro, Pakistan

*European Journal of Medical Research* 2017, **22(Suppl 1):**P7

To evaluate right ventricular function after surgery for ischemic or valvular heart disease using antegrade or retrograde blood cardioplegia. This prospective comparative study included 108 consecutive patients who underwent cardiac surgery from March 2013 to October 2014 at Punjab Institute of Cardiology, Lahore Pakistan. Patients requiring coronary artery surgery, aortic valve replacement or combined aortic and mitral valve replacement were included. Among the 108 total patients, 38 (35.19%) patients were females. The mean age of the patients was 39.62 ± 16.15 year. 54 patients received antegrade blood cardioplegia (Group A) and the other 54 received antegrade as well as retrograde blood cardioplegia with topical ice slush (Group B). Each group contained equal number of patients (18) undergoing CABG, AVR and DVR. Right ventricular function was assessed in all patients using tissue Doppler on 2D echocardiography before and after the operation. Patients in both groups were similar in their preoperative LV and RV function, severity of coronary disease and serum creatinine. Operative data showed significantly shorter aortic cross clamp time and cardiopulmonary bypass time in group B. Time required for ventilation, ICU stay and total hospital stay were also shorter for group B patients. Postoperative echo showed significantly better RV function [postoperative RVSTDI 11.82 ± 2.24 (Group A) vs 13.93 ± 3.06 (Group B) p < 0.001] although LV function did not differ significantly. Right ventricular preservation during surgery is better accomplished with combined antegrade and retrograde blood cardioplegia than antegrade blood cardioplegia alone.

### P8 Ocular involvement in porphyria cutanea tarda

#### Asra Talpur, Khalid Iqbal Talpur

##### Sindh Institute of Ophthalmology and Visual Sciences, Hyderabad, Pakistan

*European Journal of Medical Research* 2017, **22(Suppl 1):**P8

Porphyrias are a group of inherited disorders in which absence of one or the other enzyme from heme synthesis pathway leads to build up of heme precursors called porphyrins. Accumulation of porphyrins in skin in cutaneous porphyrias causes photosensitivity due to their ability to produce free radicals which damage skin when exposed to sun. Porphyria cutanea tarda is commonest form of cutaneous porphyrias. Ocular involvement in porphyria cutanea tarda is an overlooked aspect of the disease. Porphyria cutanea tarda mainly involves the exposed parts of eye namely lids, cornea, conjunctiva and sclera. Ocular manifestations include Lid scarring and subsequent cicatricial ectropion, epiphora secondary to Lacrimal scarring, corneal scarring or melting, and Scleromalacia perforans, a rare form of scleritis which may be sight threatening due to Globe perforation. We put forth 2 cases of porphyria cutanea tarda which presented with ocular involvement highlighting the importance of ophthalmic opinion in management of such patients

### P9 Clinical and hematological profile of acute myeloid leukemia (AML) patients of Sindh

#### Farzana Chang^1^, Tahir Sultan Shamsi^2^, Ali Muhammad Waryah^1^

##### ^1^Liaquat University of Medical & Health Sciences, Jamshoro, Pakistan; ^2^National Institute of Blood Diseases, Kharachi, Pakistan

*European Journal of Medical Research* 2017, **22(Suppl 1):**P9

This abstract is not included here as it has already been published [1].

Reference

1. Chang F, Shamsi TS, Waryah AM. Clinical and hematological profile of acute myeloid leukemia (AML) patients of Sindh. J Hematol Thrombo Dis. 2016;4:239.

### P10 Association of tumor viruses with disease burden in our society

#### Furqan Ahmed Bhatti

##### Department of Pathology, Liaquat University of Medical and Health Sciences, Jamshoro, Sindh, Pakistan

###### **Correspondence:** Furqan Ahmed Bhatti

*European Journal of Medical Research* 2017, **22(Suppl 1):**P10

Tumor or oncogenic viruses have the ability to induce transformation/oncogenes in the host genes. This group includes a number of common human pathogens like hepatitis B virus (HBV) and Hepatitis C virus (HCV), Epstein Barr virus (EBV), human papilloma virus (HPV). The aim of this study is to look at the burden of various conditions associated with tumor viruses. Retrospective and Descriptive study done at the Department of Pathology, Liaquat University of Medical and Health Sciences, Jamshoro. A retrospective analysis of histopathological data was performed from August 2013 till December 2013. Patients diagnosed with tumor virus-related conditions, both inflammatory and malignant, were included in this study. A total of 2723 patients attended the hospital laboratory during this period. 174 patients presented with cervical lesions such as chronic cervicitis (159 cases), cervical polyp (09 cases) and squamous cell carcinoma of cervix (06 cases), and all were related with HPV. Chronic hepatitis was diagnosed in majority of liver samples (43 out of 45) while the remaining 2 were labeled as hepatocellular carcinoma. Both liver specific viruses like HBV and HCV were mainly responsible. 07 patients had Hodgkin’s lymphoma while 02 had nasopharyngeal carcinoma and all were supposed to be related with EBV. The current data on conditions especially cervical lesions suggest a high prevalence of tumor viruses in our population. Patients diagnosed with chronic cervicitis may have underlying neoplastic changes suggesting an imperative need to develop and practice effective screening as well as diagnostic methods for these common human pathogens.

### P11 Constriction band sequence along with associated malformations

#### Sadia Effendi

##### Department of Anatomy, LUMHS, Jamshoro, Pakistan

###### **Correspondence:** Sadia Effendi

*European Journal of Medical Research* 2017, **22(Suppl 1):**P11

This abstract is not included here as it has already been published [1].

Reference

1. Effendi S, Goswami P, Kumar M. Constriction band sequence along with associated malformations. IOSR J Dent Med Sci. 2013;7(6):56–61.

### P12 To assess anti diarrheal measures taken by caretakers for their children aged < 5 years and their association with occurrence of diarrhea

#### Fahad Ahmed Memon, Khalida Naz Memon, Pashma Memon, Gulzar Usman, Bilal Razzaq Memon

##### Department of Community Medicine, LUMHS, Jamshoro, Pakistan

*European Journal of Medical Research* 2017, **22(Suppl 1):**P12

A community based descriptive cross-sectional study of 3 months’ duration was conducted on two hundred children of age ≤ 5 years residing in a slum area of Hyderabad. Information regarding occurrence of diarrhea among them as well as diarrhea protective measures adopted by care-takers were collected on a questionnaire. The care-takers of children were the responders. The data were analyzed in SPSS version 16.00 by computing frequency and percentage for all variables of interest and odds of occurrence of diarrhea in relation to anti-diarrheal practices were interpreted. Among 200 children of age less than 5 years residing in one hundred and fifty houses, 145 (72.5%) children with mean age 1.98 ± 0.86 years were suffering from diarrhea. Around 50% children were having frequent diarrheal episodes. Although 51% of the subjects in our study were using chlorinated piped water, still found the odds of occurrence of diarrhea for consuming un-boiled water, storing water and non-covering drinking water were 2.8 (955 CI 1.41–5.63; p = 0.003), 2.9 (95% CI 1.31–6.8; p = 0.009) and 3.7 (95% CI 1.5–8.8; p = 0.003) respectively. There were 85 (42.5%) of mothers who had no idea about optimum time of starting weaning food to their babies; another found 107 (53.5%) care givers had no idea about general danger signs related with diarrhea. Diarrhea protective measures were not being practiced at the optimum level despite of being highly cost-effective measures in a less privileged population, resulting in higher rate of occurrence of diarrhea among children.

**Keywords:** Diarrhea, Anti-diarrheal measures, Danger signs, Hygiene.

### P13 To determine the clinico-hematological findings of pure red cell aplasia in patients at diagnostic & research laboratory, Hyderabad

#### Faheem Ahmed Memon^1^, Ikramuddin Ujjan^2^

##### ^1^Department of Pathology, LUMHS, Jamshoro, Pakistan; ^2^Diagnostic and Research Laboratory, LUMHS, Hyderabad, Pakistan

*European Journal of Medical Research* 2017, **22(Suppl 1):**P13

Pure red-cell aplasia is a rare type of anemia or disorder that can be either idiopathic or associated with certain autoimmune diseases and affecting precursors of only red blood cells. It is a syndrome characterized by normochromic, normocytic anemia, reticulocytopenia (< 1%), and an almost complete absence of erythroblasts (< 0.5%) from the bone marrow. To study the clinical & hematological findings of pure red cell aplasia in patients. Cross Sectional Descriptive Study. It was carried out at Diagnostic & Research Laboratory LUMHS Hyderabad from October 2014 to September 2016. Detailed history, Clinical and hematological findings were recorded on a pre-designed Performa. Patients with normochromic normocytic anemia, low reticulocyte count and absent erythroblasts from the bone marrow were labeled as pure red cell aplasia. Pure red cell aplasia was diagnosed in 5 patients/children from age 8 months to 8 years with male female ratio of 2:3. Fever, weakness and anemia was present in all cases, Reticulocyte count was 0.6 & 1% in 2 patients while in 3 patients there was reticulocytopenia, ME Ratio was recorded from 15:1 to 25:1 that shows reduced erythropoiesis in bone marrow examination in all cases. Pure red cell aplasia is a type of anemia present in children.

**Keywords:** Pure red cell aplasia, Anemia, Reticulocytopenia, Erythroblasts.

### P14 Comparative study on social and emotional impact of acne in male and female in teen age attending dermatology out patient department LUMHS

#### Faiza Memon, Aneela Atta Ur Rahmaan, Muhammad Ilyas Siddiqui

##### Faculty of Community Medicine & Public Health Sciences, LUMHS, Jamshoro, Pakistan

*European Journal of Medical Research* 2017, **22(Suppl 1):**P14

Acne, is a public health issue affect 85% of teenagers between 11 and 19 years of age. The objectives of this study is to document the prevalence and psychosocial and emotional impact of acne in either gender age between 11 and 19 years. The cross sectional, comparative study was carried out at outpatient of Dermatology Department Liaquat University Hospital Hyderabad. The data was collected through a structured performance and detailed information related to acne was collected. Total 117 (33.4%) males and 233 (66.6%) females were selected. Study shows that 64.9% females suffered from light-moderate acne and 75.4% suffered from severe acne. In contrast, males who suffered from light-moderate and severe acne were 35.1 and 24.6%, respectively. Only 1.4% subjects belonged to upper socioeconomic status. Result showed that acne interferes with daily social life/events of the respondents, statistically association with acne was significant (OR = 2.87, 95% CI 1.24–6.64) as compared to those who have a tendency to feel otherwise. 64.2% of the respondents showed a positive family history of acne. Result showed that participants were bullied by people as opposed to those who did not have acne and had significant association between people with acne (OR = 3.69, 95% CI = 1.87–7.27, p value 0.03). Teenagers with acne were three times more prone to face discrimination (OR = 2.58, 95% CI 1.32–5.05, p value = 0.004) as opposed to those without acne. This study shows that women suffer from emotional and psychosocial disturbances due to Acne at a higher extent than men.

### P15 Risk factors for intrauterine fetal deaths at Liaquat University of Medical and Health Sciences

#### Faiza Shabir Ahmed, Feriha Fatima

##### Liaquat University Hospital, Jamshoro, Pakistan

*European Journal of Medical Research* 2017, **22(Suppl 1):**P15

Intrauterine fetal death refers to babies with no signs of life in utero. Intrauterine fetal death is a significant contributor to perinatal mortality. Identifying the causes and risk factors for death may aid its prevention. This study was conducted to determine the risk factors and etiology of intrauterine fetal death. A Cross-sectional study was conducted at the Department of Gynae and Obs. Unit-1, LUMHS, Jamshoro from 1st April 2015 to 30th September 2015. All pregnant women diagnosed as singleton intra-uterine fetal death were included in study after verbal informed consent. Different risk factors were examined such as age, previous medical and obstetric records, gestational week and body mass index. There were 30 cases of singleton intra-uterine fetal death during that period. Mean age was found to be 29 years. Out of 30 women 38% admitted with complaints of reduced fetal movements, 30% with fits, 24% with per vaginal bleeding and 8% with raised blood pressure. 54% of women had previous bad obstetrical history whereas 10% of women had preeclampsia in previous pregnancies. Causes of intrauterine fetal death in our study was found out to be 31% preeclampsia and eclampsia, 30% placenta abruption, 15% obstructed labour, 8% cord accidents whereas in 16% of the women, we could not determine the etiology. The risk factors for intrauterine fetal death in our study appear preventable. Emphasis should be given on antenatal care, identification of high risk cases and their proper management.

**Keyword:** Intrauterine fetal death, Risk factors causes.

### P16 Potential prevention of methamphetamine induced neurological alteration by Nigella sativa in male albino mice

#### Farhana Rajpar, Samreen Memon, Pushpa Goswami

##### Department of Anatomy, LUMHS, Jamshoro, Pakistan

*European Journal of Medical Research* 2017, **22(Suppl 1):**P16

Methamphetamine (MA) is drug of illicit, widely used as psycho stimulant due to its attention-enhancing effects. *Nigella sativa* (NS) is a plant which has been used as folk medicine since centuries. This study was aimed to determine protective NS against MA induced neuronal and behavioral changes in male albino mice. This study was conducted at Anatomy Department, Liaquat University of Medical and Health Sciences (LUMHS), Jamshoro. 60 mice were selected and were randomly divided into four groups with 15 animals in each. Group A (Control), group B (MA treated mice), group C (NS treated mice and group D (NS + MA). Behavioral tests were performed. Brain was dissected out at the end of study period and sections were stained with H & E for microscopic examinations. Data was analyzed using SPSS version 22. Continuous variables were analyzed using Student’s t test and ANOVA. The categorical variables were analyzed using Chi square test. Weight of mice was equal in all groups, while at the end of study period, weight was reduced in group B (p < 0.001). Animals in group B showed hyperactivity (distance covered was found maximum (p = 0.001). histological findings in this group showed astrocytosis (hypercellular glial tissue), and necrosis in different parts of hippocampus. *Nigella sativa* treated mice revealed intact tissue architecture and normal cellularity and was comparable to control. Neuroprotective effects of NS against MA induced gross morphological and histological alterations were confirmed.

**Keywords:** Methamphetamine, *Nigella sativa*, Hyperactivity, Histomorphology.

### P17 Correlation between malondialdehyde, coenzyme Q10 and magnesium in patients with pre-eclampsia

#### Farheen Shaikh^1^, Muhammad Yousuf Memon^1^, Tazeen Shah^2^, Shafaq Ansari^3^

##### ^1^Department of Biochemistry, Liaquat University of Medical and Health Sciences, Jamshoro, Sindh, Pakistan; ^2^Department of Physiology, Liaquat University of Medical and Health Sciences, Jamshoro, Sindh, Pakistan; ^3^Department of Anesthesiology, Liaquat Medical Hospital, Jamshoro, Sindh, Pakistan

*European Journal of Medical Research* 2017, **22(Suppl 1):**P17

The pre-eclampsia is most common hypertensive disorder during pregnancy. During oxidative stress Malondialdehyde, Coenzyme Q10 and Magnesium has been proposed as novel factors in development of pre-eclampsia. The aim of this study was to know the correlation between Malondialdehyde, Coenzyme Q10 and Magnesium in pre-eclamptic patients. The study was conducted in Biochemistry Department and Department of Obstetrics & Gynecology, LUH Jamshoro/Hyderabad. Total 210 voluntaries were taken for the study, from which 50 voluntaries were normotensive pregnant control women. 160 were patient with pre-eclamptic as cases. Malondialdehyde was analyzed on spectrophotometer, CoQ10 was evaluated on HPLC while Magnesium on ASS. Gestational ages of controls were 28.76 ± 4.48 of and cases were observed (mean ± SD) 28.37 ± 4.49 months. Systolic blood pressure in controls was 113.20 ± 9.13 mmHg while in pre-eclamptic 175.85 ± 2.59 mmHg (p < 0.02). Diastolic B.P in controls was 74.40 ± 6.75 mmHg and pre-eclamptic subjects it was 99.71 ± 13.75 (p < 0.01). Plasma malondialdehyde levels in controls were 1.54 ± 0.28 nm/l and in pre-eclamptic subjects were 2.42 ± 0.63 nmol/l (p < 0.01). Plasma Coenzyme Q10 levels (mean ± SD) in controls were 1.08 ± 0.37 μg/l and in pre-eclamptic subjects were 0.21 ± 0.09 μg/l (p < 0.05). Serum magnesium levels in controls and pre-eclamptic women were 2.17 ± 0.87 and 1.40 ± 0.427 mg/dl (p ≤ 0.03) respectively. The serum magnesium levels were found to be lower (p < 0.03) in pre-eclamptic women compared to controls. The present study revealed up-regulation of plasma malondialdehyde causes oxidative stress which lead to deficiency of CoQ10 and magnesium in pre-eclamptic patient.

### P18 Head and neck cancer burden in South Sindh Pakistan

#### Fayaz Hussian Mangi^1^, Binafsha Manzoor Syed^2^, Jawaid Naeem Qureshi^3^, Ikramud Din Ujjan^4^, Naeem Ahmed Laghari^1^

##### ^1^Nuclear Institute of Medicine and Radiotherapy, Jamshoro, Pakistan; ^2^Medical Research Centre, Liaquat University of Medical & Health Sciences, Jamshoro, Pakistan; ^3^Departments of Surgery, Liaquat University of Medical & Health Sciences, Jamshoro, Pakistan; ^4^Departments of Pathology, Liaquat University of Medical & Health Sciences, Jamshoro, Pakistan

*European Journal of Medical Research* 2017, **22(Suppl 1):**P18

The aim of this study was to assess the burden of cancer of Head Neck in south Sindh. The data was retrospectively collected from Nuclear Institute of Medicine and Radiotherapy (NIMRA) Jamshoro from prospectively established Institutional database from 2008 to 2014. For this study the patients presented with Head Neck cancers. Data was analyzed using SPSS Version 21.0. Out of 15,906 patients presented with cancers 27.84% (n = 4428) patients had cancer of head and neck. Among these patients males were 2627 (59.3%) and females were 1794 (40.7%). Carcinoma of cheek was the most common site in both sexes (37.38% in males, 30.12% in females), the second common cancer in males was larynx (14.37%) whereas in females was tongue (16.56%). The third common carcinoma in males was tongue (14.12%) and in females it was thyroid (6.69%). In males fourth and fifth common cancers were oral cavity (5.52%) and lip (3.31%). In females, the fourth and fifth carcinomas were oral cavity (5.58%) and pharynx (4.29%) respectively. Burden of the head and neck cancers is more in our population where a considerable number presents with cheek and oral cavity related cancer which is contrary to the other populations. Betal nuts chewing and usage of tobacco is highly suspected causes. However further studies are required to understand causal relationships as well as genetic and biological pattern.

### P19 Epidemiology of burn injuries among patients admitted in a tertiary care hospital in Pakistan

#### Fiza Shah Syed, Madiha Shah, Sanam Pahnwar

##### Liaquat University Hospital, Hyderabad, Pakistan

*European Journal of Medical Research* 2017, **22(Suppl 1):**P19

To assess patterns of admissions and burns among patients reporting to a tertiary care hospital in Hyderabad, Sindh, Pakistan. A retrospective chart study was conducted involving data from the Burns Ward, Liaquat University Hospital, Hyderabad which reviewed all patients admitted to the ward between January 2013 and 15th October 2016. Additionally, a 1-year review of all outpatients in the Burns Ward (October 2015 to October 2016) was performed to supplement the results from the 4-year patient database. A total of 2081 patients were admitted during the 4-years study period whereas 3000 patients were treated as outpatients during the preceding 1 year. There was a male dominance with 1155 male patients (55.5%) and 927 females (45.5%). The patient age ranged from neonates (2 days old) to 95 years. The 3 most common burns were Thermal (957 cases, 58.4%), Scalds (313 cases, 19.1%), and Electric (290, 17.7%). The TBSA ranged from 0.5 to 100%, with an average TBSA of 23% and a median TBSA of 17% (IQR = 10–30%). The relationship between TBSA and patient end outcome was found to be significant (p < 0.001). There was no significant association found between patient gender with TBSA% (p = 0.9) nor with patient outcome (p = 0.8). However, there was a significant relationship between patient age at admission and patient end outcome (p = 0.03).1019 patients (48.9%) were discharged after treatment, 81 patients (3.9%) were referred for advanced medical care, 130 patients (6.3%) left against medical advice (LAMA), and 704 patients (33.8%) were discharged upon request. Overall, the mortality within the study sample was 5.7% (n = 119).

### P20 Effects of automobile exhaust fumes on systemic inflammatory markers

#### Hina Riaz^1^, Binafsha Manzoor Syed^2^, Zulfiqar Laghari^3^, Suleman pirzada^2^

##### ^1^Department of Physiology, LUMHS, Jamshoro, Pakistan; ^2^Medical research centre, LUMHS, Jamshoro, Pakistan; ^3^Sindh University, Jamshoro, Pakistan

*European Journal of Medical Research* 2017, **22(Suppl 1):**P20

Pakistani urban territory reportedly has the highest air pollution in South Asian region. Air pollution has both short term and long term effects on human health, the effects are cast upon a variety of different organs and systems ultimately reaching down to the cellular level. This study aims to assess the effect of automobile exhaust fumes on systemic inflammatory markers including C-reactive protein and leukocytes count, IL-6, IL-13, IL-8, TNF-α and TNF-β in healthy automobile vehicles drivers and hostel resident students. Eighty-seven non-smoking, healthy drivers and same number of students were recruited for this study, their C-reactive protein concentration and leukocytes count were analyzed. Care was taken that automobile vehicle drivers must have daily exposure of at least 5 h; further they were categorized into two groups, first who have been driving for 5 years, second that have been driving for more than 5 years. Their blood samples were analyzed for IL-6, IL-13, IL-8, TNF-α and TNF-β. C-reactive protein of drivers and students was (0.36 vs. 0.31, p = 0.003) and total leukocytes count were (8.55 vs. 8.06, p = 0.04). Whereas systemic inflammatory markers levels were; IL-6 (29%), IL-8 (22%), IL-13 (17%) TNF-α (37%) and TNF-β (29%). Results of this study suggests that daily exposure of automobile exhaust fumes causes increase in inflammatory markers of apparently healthy automobile vehicles drivers.

### P21 Screening of common variants, W24X and W77X in GJB2 gene of non-familial hearing impairment in Pakistani population

#### Hina Shaikh

##### MBGD, LUMHS, Jamshoro, Pakistan

###### **Correspondence:** Hina Shaikh

*European Journal of Medical Research* 2017, **22(Suppl 1):**P21

Hearing impairment is the most common inherited neurosensory disorder and is genetically and clinically heterogeneous in nature. It has an overall prevalence of 1 in 1000 newborns whereas in Pakistan it is 1.6 per 1000 and GJB2 is the frequently mutated gene in deafness worldwide and in Pakistan.

**Purpose/objective:** This study was aimed to identify the role of common mutations of GJB2 gene in sporadic patients with congenital hearing impairment in our population. The study was comprised of the enrolment of the sporadic cases (only single affected individual) with congenital hearing impairment. After obtaining informed consent, 50 sporadic cases were enrolled. A standard pure tone audiometry was performed to evaluate the hearing threshold. Peripheral blood was obtained and genomic DNA was extracted by using standard protocol non-organic (proteinase k method), for mutation screening and sequencing. The family history showed that all the affected individuals were congenitally deaf without any family history. The audiometric screening revealed that all affected were severe to profound deaf. The sequencing analysis of GJB2 gene revealed the common homozygous mutations c.71G>A (p.W24X) and c. 231G>A (p.W77X) in the two affected individuals (4% 2/50). While there was no GJB2 mutation in remaining 48 samples.

**Conclusions:** The study indicates the role of GJB2 mutations in non-familial deaf cases. The present of common stop gained mutations of GJB2 gene in only 4% of deaf participants indicate the genetic heterogeneity of the population. This finding may help to provide genetic counseling and carrier screening for the management of hearing impairment.

### P22 HCV prevalence and awareness in students University of Sindh Jamshoro

#### Shaikh Jeeaindo^1^, Hidayatullah Mahesar^1^, Naem Tarique Narejo^2^

##### ^1^Department of F.W.B & Fisheries, University of Sindh, Jamshoro, Pakistan; ^2^Department of Physiology, University of Sindh, Jamshoro, Pakistan

*European Journal of Medical Research* 2017, **22(Suppl 1):**P22

To determine the prevalence of Hepatitis C-infection, risk factors and awareness in Students University of Sindh, Jamshoro. The participants were invited to attend the free screening camp of HCV in the Department of Physiology university of Sindh Jamshoro; The blood sample of 3 ml was taken by venipuncture from the volunteers, serum is separated through centrifuge on 5000 rpm for 10 min and then serum was tested on one step-HCV device to find anti-HCV in the serum and confirmed by ELISA. The total N = 704 volunteers participated in this study whereas males were 391 (55.5%) and females were 313 (44.5%). The age range was from 18 to 30 years, the mean age was 21.32 years. The 22 years aged volunteers were found more affected n = 12 (54.5%) comparatively to others. The infected subjects were found n = 22 (3.10%) and negative were n = 682 (96.90%). According to gender infection was found n = 12 (3.80%) in females n = 10 (2.60%) in males. Students concerned 545 (79.90%) know about hepatitis and 137 (20.10%) don’t know the hepatitis who were not infected with HCV, in infected students the knowledge of HCV was found 20 (90.90%) while 2 (9.10%) were unaware. The awareness amongst males was 10 males and 12 females were aware about HCV infection. Females were found little bit more affected than males it may be due to having no knowledge of risk factors and routes of spread of HCV infection.

### P23 Evaluating the risk of low Apgar score among new borns delivered to consanguineous parents: a hospital based study

#### Khalida Naz Memon, Aneela Atta Ur Rahman

##### Department of Community Medicine, LUMHS, Jamshoro, Pakistan

*European Journal of Medical Research* 2017, **22(Suppl 1):**P23

The conventional Apgar scoring is the rapid assessment method of clinical condition of the new borns at earliest minutes of life. The effect of consanguinity on Apgar scores of new borns has been least researched in our set up. To investigate the association between various degrees of parental consanguinity & Apgar score at birth. A comparative cross sectional study was conducted on 879 new borns delivered in various hospitals by filling a pre designed questionnaire. Parental consanguinity and Apgar score ≤ 6 at 1 and 5 min were the selected variables for the study. The results were compiled through bivariate & multivariate logistic regression analysis. The mean Apgar at birth among consanguineous & non-consanguineous new borns were 6.87 ± 0.97 and 7.65 ± 0.73 respectively. The same in total inbred new borns was recorded as 6.67 ± 0.94. After controlling for selected variables, the new borns delivered to first degree consanguineous parents were at higher risk of being born with Apgar score ≤ 6 at 1-min (OR 9.14, 95% CI 4.95, 16.85; p = 0.00). The prematurity was identified as the most potential confounder in both groups of subjects (OR 13.75, 95% CI 8.30, 22.77; p = 0.00). The sub-analysis demonstrated adjusted OR for low Apgar as 14.26 (95% CI 7.10, 28.64; p = 0.00) in term babies v/s 2.52; (95% CI 0.71, 8.95; p = 0.15) in pre-terms. Parental consanguinity is a risk factor for low Apgar at birth; however more rigorous studies separately incorporating the gestational age at birth will be more conclusive.

**Keywords:** Consanguinity, Inbreeding, Apgar score, Prematurity.

### P24 Isolation of parasitic ova from different locations in Karachi: potential source of the infection through the Pakistani currency

#### Maria Jawed Badvi^1^, Jawed Ahmed Badvi^2^, Kulsoom Jawed^3^, Ikram Din Ujjan^1^, Arshi Naz^1^

##### ^1^Diagnostic and Research Laboratory, Hyderabad, Pakistan; ^2^Ghulam Mohammad Mehar College, Sukkur, Pakistan; ^3^Dow International Medical College, Karachi, Pakistan

*European Journal of Medical Research* 2017, **22(Suppl 1):**P24

This abstract is not included here as it has already been published [1].

Reference

1. Badvi JA, Jawed K, Badvi MJ. Parastical ova isolated from the various locations and are the potential source of the parasitic infection through the Pakistan currency. Int J Vaccines Vaccin. 2016;3(2): 00061.

### P25 Gems in the cornea

#### Mohsin Iqbal Haroon

##### S.I.O.V.S, Hyderabad, Pakistan

###### **Correspondence:** Mohsin Iqbal Haroon

*European Journal of Medical Research* 2017, **22(Suppl 1):**P25

Cystinosis is an autosomal recessive disorder that results in accumulation of cysteine in most body tissues. A defect in lysosomal cysteine transport allows cysteine to be deposited with in lysosomes of the cells. Cystinosis can present as nephropathic and non nephropathic forms. Both the forms present with corneal crystalline deposits initially in the anterior peripheral stroma then precedes posteriorly and centrally, these deposits cause severe photophobia and recurrent erosions. The crystals may be deposited in the conjunctiva and retina. Cysteine deposition takes place in other organs like kidneys, thyroid, spleen, testes which presents with polyuria, polydipsia ultimately renal failure; hypothyroidism; splenomegaly, infertility. Diagnosis is made by the presence of an elevated leukocyte cysteine level and/or evidence of crystal formation in the cornea. Treatment with cysteine depleting drug; cysteamine should be initiated as soon as possible and continued lifelong to prolong renal function survival and protect extra renal organs. This talk will highlight a rare case of corneal cystinosis.

### P26 Microscopic changes produced by acrylamide in breast tissue of adult female mice

#### Nadia Khan, Samreen Memon

##### Department of Anatomy, LUMHS, Jamshoro, Pakistan

*European Journal of Medical Research* 2017, **22(Suppl 1):**P26

Acrylamide is formed when carbohydrate rich diet heats at high temperature > 120 °C. It is also present in protein rich diet, coffee, cigarette smoking and cosmetics. Its relationship with cancer has been discovered in recent years. This study was conducted to examine the association of dietary acrylamide with morphology of breast tissue. This experimental study was carried out at Anatomy Department Liaquat University of Medical and Health Sciences Jamshoro and Animal house Sindh Agriculture University Tando Jam from September 2015 to February 2016. 60 adult female albino mice of average weight 25–30 g were divided into three groups (Group A control = 20; group B 0.1 μg Acrylamide = 20; group C 0.5 μg Acrylamide = 20. Acrylamide was prepared in drinking water and was given throughout the study period. Animals were weighed on weekly basis and were examined grossly for toxic effects. Mice were sacrificed by cervical dislocation and breast tissue was taken as tissue strips from cervical to inguinal region. Tissue was processed for H&E staining and microscopic examination. Animals of group A and B were observed to be lethargic after first week of drinking acrylamide as compared to control group. Significant difference was observed in weight of mice after acrylamide (0.5 μg) intake (p < 0.02). Histologically, acrylamide treated mice have significantly increased adipose tissue and less breast stromal tissue in their breast p < 0.002. This animal based study showed a positive relation between acrylamide intake and alteration in composition of breast tissue.

**Keywords:** Acrylamide, Breast tissue, Adipose tissue.

### P27 Gynecological malignancies: still a great challenge

#### Naheed Perveen

##### Department of Gynaecology & Obstetric Unit IV, Liaquat University of Medical and Health Sciences, Jamshoro, Pakistan

###### **Correspondence:** Naheed Perveen

*European Journal of Medical Research* 2017, **22(Suppl 1):**P27

The purpose of present study is to observe the frequency and Spectrum of different types of Gynecological malignancies. This study was conducted at the Department of Gynaecology & Obstetric unit IV, Liaquat University of Medical and Health Sciences Jamshoro, during period from January 2014 to December 2015. In which all the patients with Gynecological malignancies, admitted in Department of Gynaecology & Obstetric unit IV, either diagnosed clinically, radiologically or surgically are included in this study. All benign tumors are excluded. A predesigned proforma will be filled from the case notes of admitted patients to extract the relevant data like, age, marital status, parity, religion, education, socioeconomic status, clinical presentation, tumor site, stage of disease (clinical, surgical) and surgical procedure. The diagnosis and type of malignancy has been confirmed on the histopathology report of the specimen taken. We reported total 65 patients with different gynecological malignancies. Among these 38% (25) women were with ovarian malignancy, almost equal number 37% (24) cases were with cervical cancer, 12% (08) patient were with Uterine malignancy, carcinoma of Vulva was reported in 6% (4) patients, cancer of vagina in 5% (3) cases one patient was with choriocarcinoma and one with immature mole 1.5% were observed. Gynecological malignancies are common in developing countries like us, still women are dying is a great challenge: it is needed to increase awareness programs among the populations, strengthen our screening programs, make it easy and cost effective for all the population.

### P28 Prevalence of transfusion transmissible infections in blood donors of Pakistan

#### Naveena Fatima, Aisha Arshad, Munira Borhany, Nida Anwar, Imran Naseer, Rehan Ansari, Samson Boota, Mustansir Zaidi, Tahir Shamsi

##### Department of Blood Bank of National Institute of Blood Disease and Bone Marrow Transplantation (NIBD), Karachi, Pakistan

###### **Correspondence:**

*European Journal of Medical Research* 2017, **22(Suppl 1):**P28

This abstract is not included here as it has already been published [1].

Reference

1. Arshad A, Borhany M, Anwar N, Naseer I, Ansari R, Boota S, Fatima N, Zaidi M, Shamsi T. Prevalence of transfusion transmissible infections in blood donors of Pakistan. BMC Hematol. 2016;16:27.

### P29 Spectrum of haematological disorders on bone marrow examination

#### Nazia Hafeez, Ikramdin Ujjan, Faheem A. Memon, Arshi Naz

##### Diagnostic and Research Lab, Jamshoro, Pakistan

*European Journal of Medical Research* 2017, **22(Suppl 1):**P29

Hematological disorders include a wide range of diseases from nutritional anemia to hematological malignancies and are quite frequent in all age groups. Bone marrow examination is a simple, reliable and effective technique in the diagnosis of various haematological and non-haematological conditions by providing reliable information regarding bone marrow cellularity, architecture, the stage of maturation of different blood cells and presence of any extramedullary/abnormal cell. The study was aimed to analyze the spectrum of hematological disorders diagnosed on bone marrow examination. Retrospective study. Laboratory reports of patients who underwent bone marrow examination (aspiration/biopsy) at the Diagnostic & Research Laboratory, Hyderabad. From Oct 2014 to Oct 2016. A total of 324 patients were included in this study, mean age was 23 years (2 months–75 years). 189 (58.3%) males and 135 (41.6%) were female. Out of 324 patients, 103 (31.7%) had normal and 221 (68.2%) had pathological bone marrow. In younger patients the commonest disorders were acute leukemia (10.4%), followed by Aplastic anemia (9.56%) and ITP (7.09%). In old age group the most frequent disorders were Chronic leukemia (5.24%) followed by Non Hodgkins lymphoma (1.5%) and multiple myeloma (1.23%). Disorders that were common in both age groups were megaloblastic anemia (20.06%), followed by mixed deficiency anemia (3.39%). Megaloblastic anemia was the most frequent nonmalignant hematological disorder whereas, Acute leukaemia was the commonest hematological malignancy.

**Keywords:** Acute leukaemia, Megaloblastic anemia, ITP, Multiple myeloma.

### P30 Depression among Thari women due to drought at Bodhesar Village Nagarparkar Thar

#### Parveen Akhtar^1^, Zanab Khatoon^2^, Miss Vectoria^2^, Ghulam Abass2

##### ^1^Faculty of Community Medicine & Public Health Sciences, LUMHS, Jamshoro, Pakistan; ^2^People Nursing School, Jamshoro, Pakistan

*European Journal of Medical Research* 2017, **22(Suppl 1):**P30

Drought is a major stressor constantly affecting all over the desert area since last 3 years. It is a major risk factor which contributed in many physical and mental illness & depression is one of them. Depression is major public health issue that affecting all ages and both gender but female are more prone. To identify the depression due to drought among Thari women at Bodhesar village. Descriptive Cross Sectional study was conducted at the village Bodhesar Nagarparkar. During study 120 women were examined for depression due to drought through structured interviews. We focused on the behavior and activities of women according to Diagnostic Statistical Manual of Mental Disorders V (DSM-V) and assessed the depressive symptoms. Beside that we also addressed anxiety, arthritis, and anemia. Out of 120 respondents, the mean age was 43 years, according to DSM IV, 54 participants met the criteria of moderate depression. They reported hopelessness, helplessness, decrease sleep, fatigue or lose of energy, and worthlessness. 16 met the criteria of major depressive disorder and 20 female indicated moderate anxiety disorder, and remaining all subjects reported anxiety due to the drought in Tharparkar prevailing for a third consecutive year. Pakistan faces frequent drought in last few years at Tharparkar. Thari women are suffering from depression and anxiety due to this uncertain situation. There is need to pay attention on mental health of Thari women which is most neglected aspect in the area. The health professional should set new cost effective strategies to deal with the issues of Thari women.

**Keywords:** Depression, DSM IV, Anxiety, Hopelessness.

### P31 Case of toxoplasmosis chorioretinitis presenting as acute iridocyclitis with raised IOP

#### Rafeen Talpur

##### SIOVS, Hyderabad, Pakistan

###### **Correspondence:** Rafeen Talpur

*European Journal of Medical Research* 2017, **22(Suppl 1):**P31

Toxoplasmosis gondii is an obligate, intracellular parasite and is the commonest cause of retinochoroiditis and posterior uveitis. It usually manifest between the 2nd & 4th decades of life. It may present with blurred vision, floaters, pain, red eye, metamorphopsia and photophobia. Toxoplasmosis usually presents with unifocal superficial necrotizing retinochoroiditis. Rarely may it present with iridocyclitis, papillitis or deep retinitis. We present a case of a 30 year old female presenting with iridocyclitis and raised IOP which we diagnosed as Toxoplasmosis chorioretinitis. She was successfully managed and her VA improved from counting finger to 6/18 and IOP was within normal limits.

The author received written informed consent to publish from the patient.

### P32 Glycemic control is one of the preventive measurement from retinopathy in the patients of type-1 diabetes mellitus

#### Rafiq Ahmed, Roohi Naz, Ali Raza Memon, Zainab Memon

##### Department of Biochemistry, LUMHS, Jamshoro, Pakistan

*European Journal of Medical Research* 2017, **22(Suppl 1):**P32

Diabetic retinopathy is one of the main complication of both types; type 1 & type 2 diabetes mellitus. HbA1c is the gold parameter to estimate the glycemic index nowadays. Poor glycemic index means high blood sugar level which is the proceeding factor for development of Retinopathy. Total 80 subjects were included in the study, from which 40 were type 1 diabetes without retinopathy & 40 were diabetes type 1 with retinopathy. HbA1c was estimated by Bio Red variant. retinopathy was assessed by indirect ophthalmoscope. The mean of HbA1c type 1 diabetic patients without retinopathy was 9.87 ± 0.85% and in type 1 diabetes mellitus with retinopathy as, 11.27 ± 0.77% respectively. These results showed that glycemic control in type 1 diabetes with retinopathy highly poor than type 1 diabetic patients without retinopathy. Poor glycemic index take a part in proceeding diabetic complications like retinopathy.

**Keywords:** Type 1 diabetes mellitus, diabetic retinopathy, HbA1c.

### P33 Fetomaternal outcome of breech vaginal delivery versus planned caesarean section

#### Raheela Munwar, Sajida Rajpar, Fareen Memon, Miss Bilquees, Razia Shoukat

##### Department of Gynecology & Obstetrics, Indus Medical College Hospital Tando Muhammad Khan, Sindh, Pakistan

*European Journal of Medical Research* 2017, **22(Suppl 1):**P33

To evaluate the effect of breech vaginal delivery on feto maternal outcome in comparison of planned cesarean section. This study was carried out at Department of Gynecology and with singleton breech presentation admitted in this hospital at and after 37–41 weeks of gestation. The incidence of breech was 3%. Out of 118 singleton term breech deliveries 73 were multiparous selected for study. Parity distribution according to data was 38% primipara and 62% multipara. The comparative results of fetomaternal outcome of breech vaginal delivery vs planned cesarean section are as given below: perinatal mortality (7.2% vs 0%), > 7 Apgar score at 5 min (83% vs 85%). Obstetrical trauma (7.3% vs 0%), NICU Admission (17% vs 15%) Hospital stay > 03-days (8% vs 69%), wound infection (0% vs 7.7%) trauma to genital tract (5% vs 0%), postpartum hemorrhage (9.7% vs 15%), anesthesia complications (0% vs 7.6%). While in emergency cesarean section perinatal mortality (10.5%), Apgar score > 7 at 5 min (53%), Obstetrical trauma (5.2%), NICU admission (47%), Hospital stay > 03-days (89.5%), Wound infection (15.7%), Trauma to genital tract (21%), postpartum hemorrhage (26%) and complications of anesthesia (15.7%). The study concluded that planned breech vaginal birth in multiparous women at term is safe and reduces maternal morbidity, perinatal mortality and immediate neonatal outcome of breech vaginal delivered babies can be improved by early booking, careful case selection and vigilant labor monitoring. Planned cesarean section is relatively safe for breech babies but breech cesarean delivery either elective or emergency increases the risk of maternal morbidity.

### P34 Clinico-pathological analysis of pancytopenia in tertiary care hospital

#### Sadia Abbasi, Ikram Ud Din Ujjan, Faheem Memon, Arshi Naz

##### Diagnostic and Research Laboratory, LUMHS, Hyderabad, Pakistan

*European Journal of Medical Research* 2017, **22(Suppl 1):**P34

Pancytopenia is a common hematological condition in clinical practice which is characterized by the simultaneous presence of anemia, leukopenia and thrombocytopenia. To determine the frequency of pancytopenia, common etiology & presentation on the basis of bone marrow biopsy. Observational. October 2014 to September 2016. Medical records, blood smear, bone marrow aspirate and biopsy findings of patients presenting with pancytopenia who full fill the inclusion criteria were analyzed. Relevant history, physical, systemic examination and hematological findings at presentation were recorded using a standard proforma after approval of institutional review board and signing of informed consent of patients and their guardian. Among 97 cases of pancytopenia males 54 (55%) and females 43 (44%). The majority of the patients are falling in pediatric age group 64 (65%). Megaloblastic anemia was the most common cause of pancytopenia in 40 (41%), followed by aplastic anemia in 23 (23%), hypersplenism in 9 (9%) and leukemia in 7 (7%) cases. Megaloblastic anemia is the most common cause of pancytopenia with male predominance followed by Aplastic anemia and Hypersplenism.

**Keywords:** Pancytopenia, Megaloblastic anemia, Aplastic anemia, Bone marrow biopsy.

### P35 Clinical and haematological findings in patients of acute leukaemia diagnosed at diagnostic research laboratory Hyderabad

#### Sadia Shahmeer Qazi^1,2^, Ikram Din Ujjan^1,2^, Faheem A Memon^1,2^, Arshi Naz^1,2^

##### ^1^Diagnostic and Research Laboratory, Hyderabad, Pakistan; ^2^National Institute of Blood Disease and Bone Marrow Transplantation, Karachi, Pakistan

*European Journal of Medical Research* 2017, **22(Suppl 1):**P35

Acute leukemias (ALs) are one of the most common cancers in the world. Acute leukemias represent neoplasm of the hematopoietic cell precursors manifested as clonal expansion of myeloid and lymphoid hematopoiesis. Acute leukemia can be broadly classified into acute lymphocytic leukemia and acute myelogenous leukemia depending on the type of cell lineage affected. To analyze clinical and hematological findings in various types of acute leukemia retrospective. Diagnostic and Research Laboratory Hyderabad from Oct 2014 to Oct 2016. A total of 330 reports of bone marrow biopsy were reviewed for age, gender, diagnosis on bone marrow examination, presenting complaints and duration of illness. Thirty-one cases were diagnosed as suffering from acute leukemias, in that ALL were 18 (58%), AML 9 (29%), APML 2 (6.5%) and Burkitt lymphoma were also 2 (6.5%) cases. Male and female ratio was 2:1. Total 20 cases of ALL and 11 were reported in AML. Fever, pallor, weight loss were the main presenting complaints. The present study concludes that most common type of acute leukemia is acute lymphoblastic leukemia.

**Keywords:** Bone biopsy, Acute lymphoblastic leukemia, Acute myelogenous leukemia.

### P36 Eyelid coloboma: a study of 13 cases

#### Sama Paras, Mahesh Kumar

##### Department of Plastic & Reconstructive Surgery, L.U.M.H.S, Jamshoro, Pakistan

*European Journal of Medical Research* 2017, **22(Suppl 1):**P36

To describe our experience in the surgical management of patients with congenital eyelid colobomas in our setup. Starting and closing dates of study period with minimum and maximum follow up times. The study was conducted from January 2012 to December 2015 in the Department of Plastic & Reconstructive Surgery, L.U.M.H.S, Jamshoro. In this observational study 10 patients with different age groups of both genders having eyelid colobomas presented to the Department of Plastic and Reconstructive Surgery, LUMHS were included. Different surgical procedures depending upon the eyelid defect were used for their construction. Early intervention was done to prevent exposure keratitis and other visual impairments. Out of 10 patients that were enrolled in the study, primary closure was done in 5 patients. 4 patients underwent canthotomy and cantholysis. In 4 patients tenzel flap was used for the reconstruction of eyelid coloboma. A statement concerning the significance of the work and its possible implications for future research/modification Tenzelflap, primary closure, canthotomy and cantholysis yield good aesthetic outcomes in reconstruction of eyelid colobomas.

### P37 Clinico-haematological features of aplastic anemia

#### Sana Fatima^1^, Abdul Rehman Khalil Shaikh^1^, Faheem A Memon^1^, Ikram Din Ujjan^1^, Arshi Naz^2^

##### ^1^Diagnostic And Research Laboratory, Hyderabad, Pakistan; ^2^National Institute of Blood Disease and Bone Marrow Transplantation, Karachi, Pakistan

*European Journal of Medical Research* 2017, **22(Suppl 1):**P37

The incidence of aplastic anemia appears to be relatively high in some parts of the world, including Pakistan. Erythrocytes, granulocytes, and platelets, which are normally produced in the bone marrow, decrease to dangerously low levels. Anemia leads to fatigue, dyspnea, and cardiac symptoms; thrombocytopenia to bruising and mucosal bleeding; and neutropenia to sharply increased susceptibility to infection. The most common cause of aplastic anemia in children is Fanconi’s anemia. To study the clinical presentations in diagnosed cases of aplastic anemia. To evaluate haematological parameters including bone marrow aspiration and trephine biopsy. Retrospective Aplastic anaemia cases were evaluated clinically along with hematological parameters, bone marrow aspiration and trephine biopsy at Diagnostic & Research Laboratory, LUMHS, Hyderabad during the period of October 2014 to October 2016. Among 31 cases mean age 13 (01–30 years) and female predominance. Most of the patients presented with fever, pallor and generalized weakness. Bone marrow aspiration was hypocellular in all cases. Severe aplastic anemia was commonly diagnosed. The present study concludes that detailed primary hematological investigations along with bone marrow aspiration in pancytopenic patients are helpful for understanding disease process and to diagnose or to rule out aplastic anemia. These are also helpful in planning further investigations and management.

**Keywords:** Bone marrow aspiration, Aplastic anemia, Pancytopenia.

### P38 Impact of maternal weight on success of VBAC

#### Sana Zaheeruddin

##### Agha Khan Hospital, Hyderabad, Pakistan

###### **Correspondence:** Sana Zaheeruddin

*European Journal of Medical Research* 2017, **22(Suppl 1):**P38

Worldwide Cesarean section is the commonest obstetrical procedure to be performed and same situation is in Pakistan. One strategy is to offer vaginal birth after cesarean section to reduce the alarming cesarean rate. Many factors have been Identified which can affect success of trial of labor. Maternal weight has an important relation with the reproductive health of women, as obesity during pregnancy is associated with increased maternal and fetal risk. Maternal obesity has been shown to be associated with increased rates of primary cesarean delivery and failed trial of vaginal birth after cesarean delivery. To determine the effect of maternal weight on success of VBAC: cross sectional study: May 2012 to October 2013. Liaquat university hospital, Hyderabad. a total of 96 women which fulfilled the selection criteria were included in the study. The women included in the study had a mean age of SD (range), 29.94 + 4.41 successful vaginal births was observed in 57 (59.5%) women and 39 (40.6%) had an emergency repeat cesarean delivery. Body mass index was noted among all the women, 23 (24.0%) were obese and 73 (76.0%) were non-obese. Out of 23 (24.0), 7 (30.4%) had successful VBAC and 16 (69.6%) women had successful trial of labor and 23 (31.5%) delivered by repeat Caesarean delivery (p 0.002) *p* value = 0.001 is statistically significant and calculated by Fisher’s exact X^2^ test. Obesity is associated with decreased chances of successful VBAC, making it a risky option for obese women.

**Keywords:** VBAC, Obesity, Caesarean section.

### P39 Marital status, an independent prognostic factor for out of hospital mortality after coronary artery bypass grafting (CABG)

#### Sharjeel Abbas, Shahid Memon, Mahjabeen Shaikh, Madiha Iqbal, Waseem Riaz, Mohsin Hussain

##### Department of Sardiology, Liaquat University Hospital, Hyderabad, Pakistan

*European Journal of Medical Research* 2017, **22(Suppl 1):**P39

Marital status has been associated as an independent prognostic factor for survival in variety of tumors, for its additional social support. The aim of the study is to reflect on whether marital status has any association with mortality in patients at 1 year after post CABG hospital discharge. Computer based randomized prospective case (married) control (unmarried) study. Out patients Department of Punjab Institute of Cardiology, Lahore. From January 2010 to December 2014, a computer based randomization performed to select 100 patients in each of Group A (married) and Group B (unmarried), with ages between 35 and 70 years. CABG related survival (CRS) was compared between two groups 1 year after CABG. The mean age of the patients was 50.63 ± 15.33 years. Group A is found younger (48.36 ± 16.43 vs 55.66 ± 17.23) with decreased hospital stay (8.39 ± 4.7 vs 16.0 ± 5.84) and early recovery after CABG (98% vs 88%). CPB time and mean cross clamp time was insignificant (p > 0.05). Ninety patients were found to be asymptomatic 1 year after CABG in Group A, in contrast to 78 patients in Group B. The reported mortality was 2% in Group A whereas 12% in Group B. There is significant relationship between 1 year survival and marital status of patients who underwent CABG.

### P40 Ultra sound findings in CLD and their relation with duration of disease at LUMHS

#### Sara Khalid Memon

##### LUMHS, Jamshoro, Pakistan

###### **Correspondence:** Sara Khalid Memon

*European Journal of Medical Research* 2017, **22(Suppl 1):**P40

This abstract is not included here as it has already been published [1].

Reference

1. Memon SK. Ultrasonographic findings of liver in chronic liver disease and its complications and their association with the duration of the disease. J Coll Physicians Surg Pak. 2017;27(3):127–30.

### P41 To study the demographic and clinico-haematological findings of chronic myeloid leukemia

#### Sidra Qadir, Ikram Din Ujjan, Faheem Memon, Arshi Naz

##### Diagnostic and Research Lab, Hyderabad Jamshoro Pakistan

*European Journal of Medical Research* 2017, **22(Suppl 1):**P41

Chronic myeloid leukemia (chronic granulocytic leukemia) is a myeloproliferative neoplasm characterized by rearrangement of the long arms of chromosome 9 and 22 resulting in Philadelphia chromosome, creating the fusion oncogene BCR-ABL. To study the demographic and clinico-heamatological findings of chronic myeloid leukemia. Retrospective and observational. Diagnostic and Research Laboratory LUMHS Hyderabad. October 2014 to September 2016. 70 cases were evaluated from the records which were diagnosed with chronic myeloid leukemia at their presentation on complete blood count and Philadelphia chromosome. The common chief complaints were fever, weight loss, weakness, abdominal pain, abdominal fullness and splenomegaly. Out of 70 patients, mean age of 42 patients were 30 years (18–40) and 28 patients with mean age 50 years (40–68 years). Male female ratio was 1:1. On examination, massive splenomegaly was found in almost all patients. Laboratory parameters were anemia mean hemoglobin level 7 g/dl (4.2–12 g/dl), leukocytosis 200 × 10^3^/l (43–906 × 10^3^), 26 patients have thrombocytosis 711.8 × 10^3^/l (476–1340 × 10^3^), 11 patients have thrombocytopenia 89 (32–145 × 10^3^/l and 33 patients with normal platelets (150–450). All 70 cases were in chronic phase. Chronic myeloid leukemia is more common in young age group.

**Keywords:** Chronic myeloid Leukemia, Philadelphia, Splenomegaly.

### P42 Safety of predischarge exercise treadmil test (ETT) after different types of acute myocardial infarction

#### Sumaira Shaikh^1^, Syed Fasih Ahmad^2^, Zeeshan Nasir^3^

##### ^1^Cardiology, Liaquat University of Medical and Health Sciences, Hyderabad, Pakistan; ^2^Department Cardiology, Liaquat University of Medical and Health Sciences, Hyderabad, Pakistan; ^3^Liaquat University of Medical and Health Sciences, Jamshoro, Pakistan

*European Journal of Medical Research* 2017, **22(Suppl 1):**P42

Due to development in the prognostic information, efficacy of medical therapy, the mortality of acute myocardial infarction is significantly decreased. But the use of treadmill test as an indicator of risk had been questioned already. We can access the risk stratification by use of either treadmill ergometry test or bicycle ergometry test. Our main aim is to assess the safety of performing pre-discharge treadmill after different type of acute myocardial infarction. An observation cross sectional study that was held in cardiac Department of civil hospital Hyderabad with sample size of 41 patients with ejection fraction ≥ 40% before discharge and other inclusion and exclusion criteria. The data was collected by the help of questioner, by observing the patients during treadmill test. Response of ETT was accessed with the association of type of myocardial infarction and risk factors predisposed by calculating frequency and Chi square and bivalent test (Pearson correlation) between statistical significant entities. The mean age with 47.87 in which 36.6% Anterior MI, 56.1% inferior, 12.2% lateral MI, 14.6% posterior MI and 14.6% other types of MI patients. Association of anterior MI with Angina pain (p value 0.010), Inferior MI with Angina pain (p value 0.010) and Lateral MI with ST depression (p value 0.027) and the other results was discreet. Correlation of Anterior MI and Angina pain with weak positive identity (0.402), inferior MI and Angina Pain with weak negative identity (− 0.402) and lateral MI and ST depression with weak positive identity (0.346). Significant risk in lateral MI to perform treadmill because of ST depression and anterior MI the relation of Angina pain is much more diverse and showed a relative risk to perform treadmill and inferior MI with Angina pain is discreet and there is no much relative risk performing treadmill (required more studies). The risk of performing treadmill in posterior and others type of MI showed no risk factors, so performing tread mill will be safe.

**Keywords:** ETT (exercise treadmill test), AMI (acute myocardial infarction).

### P43 Effects of smokeless tobacco on the granulosa cells of the ovaries of non-pregnant female Swiss albino rats: a histological study

#### Syna Pervaiz Singha, Afroz Saleem Kazi, Usha Isaac

##### Isra University, Hyderabad, Pakistan

*European Journal of Medical Research* 2017, **22(Suppl 1):**P43

The use of tobacco has been linked with a higher mortality rate. Smokeless tobacco also contains higher quantities of nicotine. Low fertility rates & adverse reproductive outcomes are associated with tobacco use. An effort is made to evaluate the effects produced by the locally available brand of smokeless tobacco on the granulosa cells of the ovaries of the female Swiss albino rats. 30 adult female Swiss albino rats were randomly selected. They were equally divided into three groups. Group A were taken as control. Group B&C consisted of experimental groups which were given 5 and 10% of locally available brand of smokeless tobacco in their feed. The feed and water were given ad libitum. On 31st day, animals were sacrificed and their ovaries were removed. The specimens were processed for the purpose of light microscopy using H & E and trichrome stains. Ovaries of both B & C groups showed significant increase in the number of cystic follicles, apoptotic cell death and necrosis in the granulosa cells (p ≤ 0.001). This study concluded that the smokeless form of tobacco causes adverse effects on the granulosa cells of the ovaries of the female Swiss albino rats.

**Keywords:** Granulosa cells, Nicotine, Ovaries, Reproduction, Side effects, Smokeless tobacco.

### P44 Morphological patterns of breast cancer in females of Sindh Province: an institution based study from 2008 to 2014

#### Tanweer Ahmed Shaikh^1^, Binafsha Manzoor Syed^2^, Jawaid Naeem Qureshi^3^, Ikramdin Ujjan^4^

##### ^1^Department of Pathology, LUMHS, Jamshoro, Pakistan; ^2^Medical Research Center, LUMHS, Jamshoro, Pakistan; ^3^Department of Surgery, LUMHS, Jamshoro, Pakistan; ^4^Department of Pathology, LUMHS, Jamshoro, Pakistan

*European Journal of Medical Research* 2017, **22(Suppl 1):**P44

Globally breast cancer is the second leading and most common cancer among females effecting 1.7 million and accounting 11.9% of all cancers. Annually, 90,000 new cases are diagnosed in Pakistan and 40,000 deaths occur. This study is aimed to assess morphological patterns of breast cancer with relation to age. During the period of 7 years (Jan 2008 to Jan 2014) prospectively collected institutional data was retrieved. The patients present with breast cancer were included. Out of 15,906 patients, 2026 patients presented with breast cancer counting 12.8% of all cancer. Median age of presentation was 45 years. The predominant morphology was Infiltrating duct cell carcinoma (89.7%) and majority of the cases presented with grade-II (82.4%). Breast cancer incidence is rapidly increasing in Pakistan especially at young age. The peak incidence lies between 30 and 60 years. This pattern of presentation warrants further biological exploration of tumors so that patient be properly managed to improve the survival of the patients.

### P45 To determine the frequency of raised serum CRP level in patients with community acquired pneumonia

#### Tarachand Devrajani, Syed Zulfiquar Ali Shah, Samar Raza

##### Department of Medicine, Liaquat University Hospital, Hyderabad, Pakistan

*European Journal of Medical Research* 2017, **22(Suppl 1):**P45

This cross sectional descriptive study of 6 months study was conducted at Liaquat University Hospital Hyderabad from 04-11-2013 to 03-05-2014. All the patients with 20–75 years of age, present with fever > 38 °C, productive cough, pleuritic chest pain, dyspnea and diagnosed as community acquired pneumonia were further evaluated for C-reactive protein. During 6 month study period, total 135 patients with community acquired pneumonia were evaluated for C-reactive protein. The mean ± SD for age of patients with CAP was 48.93 ± 8.41 whereas it was 53.53 ± 6.73 and 50.54 ± 5.81 in male and female subjects respectively. The mean age ± SD of patient with raised CRP was 46.94 ± 8.43. The mean ± SD of CRP in overall population was 08.8 ± 1.52 while it was 07.94 ± 1.32 and 10.83 ± 1.64 in male and female individuals respectively. Of 135 subjects 84 (62.2%) were males and 51 (37.7%) were females while the CRP was elevated in 91 (67.4%) patients. The age in relation to gender and CRP was statistically significant (p = 0.02 and 0.05) while the gender in relation to CRP was also statistically significant (p = 0.03). The present study found that the CRP was raised in 91 (67.4%) predominantly male individuals with community acquired pneumonia.

### P46 To determine the effects of green tea on blood pressure of healthy and type 2 diabetes mellitus males

#### Tazeen Shah^1^, Farheen Shaikh^2^, Shafaq Ansari^3^

##### ^1^Department of Physiology, Liaquat University of Medical and Health Sciences, Jamshoro, Pakistan; ^2^Department of Biochemistry, Liaquat University of Medical and Health Sciences, Jamshoro, Pakistan; ^3^Department of Anesthesiology, Liaquat Medical Hospital, Jamshoro, Pakistan

*European Journal of Medical Research* 2017, **22(Suppl 1):**P46

Green tea contains more than 4000 biologically active compounds of which *polyphenols* are important. *Flavonoids* are the most important of polyphenols family. Flavonoids contain *catechins*, which are the primary polyphenols in GT. Catechins exert anti oxidant activity. Hence the GT neutralizes the free radicals produced during normal body metabolism Green tea exerts many effects on the normal physiological functions of human body. The objective/s of present study were to determine the effects of green tea on blood pressure of healthy and type 2 diabetes mellitus (DM) individuals: the present cross sectional comparative study was conducted at the Department of Physiology & Medical Research Centre, LUMHS and NIMRA Jamshoro over 6 months duration. A sample of 75 healthy controls and 75 diagnosed DM cases were selected according to inclusion and exclusion by convenient random sampling. Informed consent was taken from willing volunteers. Pulses, blood pressure, were taken as per standard protocol. Blood sugar was determined. Study was approved by ethics committee of the institute. Data was entered in Microsoft excel sheet and was analyzed on SPSS 21.0 (IBM, Incorporation, USA) Student t test and Chi square test were used for the analysis of continuous and categorical variables. Statistical significance was defined as p value of ≤ 0.05. Of 150 total study subjects, 75 were healthy control subjects and 75 were type 2 diabetics (T2DM) subjects. Pulse, systolic BP, diastolic BP, Pulse pressure, Mean arterial pressure, showed statistically significant improvement in controls and diabetics at baseline and at 90th intervention day, blood glucose was improved in controls and diabetic subjects. The present study concludes that the intake of Green tea improves pulse, blood pressure, lowers blood glucose levels. Hence, the present study concludes that the Green tea exerts beneficial effects on the cardiovascular physiology, lowering blood pressure and also blood glucose.

**Keywords:** Green tea, Blood pressure, Polyphenols, Flavonoids, Catechins, Type 2 diabetes mellitus.

### P47 Morning Larks and Night Owls; the relationship of chronotype with the intelligent quotient (IQ)

#### Urooj Bhatti, Tarim Nayab, Nehan Syed

##### Department of Physiology, LUMHS, Jamshoro, Pakistan

*European Journal of Medical Research* 2017, **22(Suppl 1):**P47

Morning larks and night owls preferring the morning and night time respectively for their major activities have been found to have different chronobiological and chronopsychological responses. This study was designed to determine the chronotype of medical students and their pattern of intelligent quotient (IQ). This cross sectional study was conducted during December 2015–September 2016 among 225 medical students from Dow University of Health Sciences (DUHS) and Liaquat University of Medical and Health Sciences (LUMHS). Informed consent was taken and students with any neurological or psychological disease were excluded. To check the chronotype we used the Lark and Owl Questionnaire and IQ was classified by Stanford Binet Intelligence Scale. Data was entered and analysed by SPSS (statistical package of social sciences) version 22. According to this study, 19% were found to be extreme evening type whereas 13% were the extreme morning type, 38% were the neutral, 23 and 5% were moderately evening and moderately morning type respectively. The evening types were seemed to have higher IQ than morning type.

**Conclusion:** Contradictory to the beliefs, evening types were found to have better cognitive function and seem dominating among medical students.

**Keywords:** Circadian rhythm, Intelligence, Students, Medical.

### P48 Study of inherited oculo-cutaneous albinism through linkage analysis in Sindhi population

#### Yar Muhammad Waryah

##### Molecular Biology & Genetic Diseases Department, LUMHS, Jamshoro, Pakistan

###### **Correspondence:** Yar Muhammad Waryah

*European Journal of Medical Research* 2017, **22(Suppl 1):**P48

Oculo-cutaneous albinism (OCA) is an autosomal recessive genetic disorder. The patients affected with OCA demonstrate a partial or total lack of melanin in the skin, hair and eye. It is caused by mutations in the TYR and OCA2 genes. The purpose of this study was to carry out genetic study of OCA in Sindhi families through linkage analysis. After informed consent, detailed family and clinical history was taken. The pedigree was drawn to determine the mode of inheritance. Genomic DNA was isolated from blood leukocytes of all the individuals included in the study. Polymerase chain reaction was performed and haplotype analysis was done for TYR and OCA locus using STR markers. Six sets of STR (short marker were used for linkage analysis of both genes. Four families affected with inherited OCA were enrolled in this study, including with 21 participants. Eleven individuals were affected with typical features of OCA such as hypopigmentation of hair and skin, blue translucent irides, nystagmus and reduced visual acuity. The linkage analysis showed one family linked with OCA2 gene and three were unlinked with both OCA2 and TYR genes. The study indicates the association of OCA2 gene with oculocutaneous albinism patients in Sindhi population. The three families were unlinked for both OCA2 and TYR gene indicate involvement of any other gene for the disease. Haplotype and clinical findings of OCA linked family may contribute for development of easy to use diagnostic method for determination of OCA2 carriers and genetic counseling of affected family.

**Keywords:** Linkage analysis, Oculo-cutaneous albinism, OCA genes.

### P49 Molecular analysis and identification of genetic polymorphisms in a patient with JAK2 negative essential thrombocythemia, unresponsive to therapy: a case report

#### Uzma Zaidi^1^, Saba Shahid^2^, Naveen Fatima^3^, Sharik Ahmed^2^, Gul Safaida^2^, Muhammad Nadeem^2^, Tahir Shamsi^1^

##### ^1^Department of Clinical Haematology, National Institute of Blood Diseases & Bone Marrow Transplantation, Karachi, Pakistan; ^2^Department of Genomics, National institute of Blood Diseases & Bone marrow Transplantation, Karachi, Pakistan; ^3^Department of Research & Molecular Medicine, National Institute of Blood Diseases & Bone marrow Transplantation, Karachi, Pakistan

*European Journal of Medical Research* 2017, **22(Suppl 1):**P49

Clonal analysis of patients with *JAK2, MPL* and *CAL*-*R* negative MPN has demonstrated the evidence of additional aberrations including epigenetic alterations, some of which have clear impact on the clinical phenotype of disease. To find out such novel genetic aberrations, patient was screened through next generation sequencing using myeloid sequencing panel of 54 genes on MiSeq. We identified genetic variants in 28 genes including *TET2*, *BCOR* and *CUX1* in a patient with triple negative ET. The individual role of these polymorphisms in disease pathogenesis has not been studied so far. Somatic mutations in the same genes have been reported with variable frequencies in myeloid malignancies. The coexistence of genetic polymorphisms in genes might be associated with poor prognosis in ET and the aggressive clinical course in this case propose the likelihood of their compound effect on the disease pathogenesis and resistance to therapy but it needs to be evaluated on large cohort of patients. Therefore, long term follow up of patients and the use of whole genome sequencing is required to assess the effects of these variants as well as to identify other genetic factors that might be responsible for this variation in clinical behavior of MPN patients.

